# Time‐Resolved Dynamics of Laser Ablation in Liquid with Gas‐Evolving Additives: Toward Molding the Atomic Structure of Nonequilibrium Nanoalloys

**DOI:** 10.1002/advs.202416035

**Published:** 2025-04-26

**Authors:** Vito Coviello, Catherine Reffatto, Mehdi W. Fawaz, Benoit Mahler, Arnaud Sollier, Bratislav Lukic, Alexander Rack, David Amans, Vincenzo Amendola

**Affiliations:** ^1^ Department of Chemical Sciences Università di Padova via Marzolo 1 Padova I‐35131 Italy; ^2^ Université Claude Bernard Lyon 1 UMR 5306 CNRS Institut Lumière Matière Villeurbanne F‐69100 France; ^3^ CEA DAM DIF Arpajon F‐91297 France; ^4^ Université Paris‐Saclay CEA Laboratoire Matière en Conditions Extrêmes Bruyères‐ Le‐Chatel F‐91680 France; ^5^ ESRF‐The European Synchrotron CS40220 Grenoble F‐38043 France

**Keywords:** laser ablation, magneto‐plasmonics, nanoalloys, nanoparticles, nonequilibrium, time‐resolved

## Abstract

Laser ablation in liquid (LAL) is a reference technique for the synthesis of multicomponent non‐equilibrium nanomaterials which have potentially disruptive properties in photonics, nanomedicine, and catalysis. Yet, ablation dynamics is poorly understood regarding the multielement matter and, therefore, the remarkable potential of LAL for controlling the local atomic structure of metastable nanophases remains largely unexploited. Here, the dynamics of LAL are investigated with non‐equilibrium gold‐iron nanoalloys generated in the presence of gas‐evolving additives, which drive the formation of different nanostructures. With analytical electron microscopy, the structure in the different conditions is properly identified through complete segregation into oxide‐metal heterostructures, precipitation of nanoclusters within the nanoalloys, or ordered solid solutions. To elucidate the unforeseen effects of the solutes on the atomic structure of nanoalloys, the early and full dynamics of LAL is investigated with time‐resolved experiments, leading to the pivotal evidence that alloying of metastable compounds with different chemical reactivity is favored by decreasing the pressure of the shockwave front. The resulting picture indicates LAL with gas‐evolving additives as a strategy for molding the atomic structure of non‐equilibrium nanoalloys, opening the way to the development of a library of advanced nanomaterials otherwise inaccessible.

## Graphical abstract

Laser ablation in liquid still holds great potential for the synthesis of multicomponent non‐equilibrium nanomaterials. Here, time‐resolved experiments reveal that gas‐evolving additives both have chemical effects and reduce shockwave pressure. This approach offers a strategy to control the atomic structure of the nanoalloys, easily switching from segregated heterostructures to metastable nanoalloys with either iron oxide or gold skin.

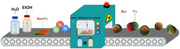

## 1 Introduction

Laser ablation in liquid (LAL) has rapidly become essential to access innovative multicomponent nanomaterials with peculiar properties, which cannot be realized with ordinary synthetic techniques.^[^ ^1^, ^2^, ^3^ ^]^ LAL starts with the absorption of laser light by a solid target immersed in a liquid solution.^[^ ^1^, ^4^ ^]^ The quick absorption of the photon beam causes the emission of two shockwaves counterpropagating from the target surface, i.e., a pressure wave travelling through the liquid and another one travelling through the target, both at supersonic speed and with energy directly related to that of the incident laser pulse.^[^ ^5^ ^]^ In a few hundred picoseconds,^[^ ^6^, ^7^, ^8^ ^]^ the ablated material results in a hot plasma containing excited atoms, clusters, and droplets of the molten target, initially confined by the liquid phase.^[^ ^9^, ^10^, ^11^ ^]^ Already a few nanoseconds after light absorption,^[^ ^7^, ^8^ ^]^ the process is followed by the generation of a cavitation bubble. In the bubble, the plasma expands and the ablated matter initiates its cooling stage, which is completed after the collapse of the bubble and the dispersion into the liquid phase when a colloid (sol) is generated.^[^ ^4^, ^5^ ^]^ The nanoparticles (NPs) are produced under out‐of‐equilibrium conditions because the entire process happens on a timescale of hundreds of microseconds for pulse energy higher than a few millijoules, during which the ablated matter experiences abrupt variations of the intrinsic and surrounding physicochemical conditions.^[^ ^4^, ^5^, ^6^ ^]^ For instance, the cooling rate can reach values of the order of 10^10^ K s^−1^ in the early 100 ns of LAL, a value unmatched by ordinary synthetic methods as the wet chemistry approaches.^[^ ^4^, ^5^ ^]^ At these extreme cooling rates, the kinetic stabilization of non‐equilibrium phases at the nanoscale is facile, as demonstrated so far with various multimetallic NPs,^[^ ^12^, ^13^, ^14^, ^15^, ^16^ ^]^ hybrid compounds,^[^ ^17^, ^18^ ^]^ amorphous oxides^[^ ^19^ ^]^ and glassy metals.^[^ ^20^ ^]^ Among them, alloy NPs made of immiscible elements play a pivotal role in venturing into the new generation of multifunctional nanomaterials.^[^ ^12^, ^14^, ^21^, ^22^, ^23^, ^24^, ^25^ ^]^ However, the ultrarapid quenching of atomic diffusion, which is achieved with non‐equilibrium routes like LAL,^[^ ^1^, ^3^, ^4^, ^5^ ^]^ is not enough to manage the differential chemical reactivity of each alloy element with the environment, especially with oxygen. The chemical transformation of non‐noble metals is a formidable challenge for the synthesis of multimetallic NPs,^[^ ^26^ ^]^ contributing to explaining why the LAL of bulk targets made of immiscible elements often leads to NPs with a different final composition. In consideration of the extreme physicochemical conditions and complex chemical processes during LAL, an inherent difficulty in predetermining the structure, morphology and final composition of non‐equilibrium nanoalloys obtained with this technique still persists.^[^ ^4^, ^21^, ^22^, ^23^, ^26^, ^27^ ^]^

Experimental and theoretical studies have indicated that the composition of the plasma,^[^ ^11^ ^]^ the dynamics of the shockwave,^[^ ^9^, ^28^ ^]^ and that of the cavitation bubble,^[^ ^29^ ^]^ all determine the final composition of the NPs.^[^ ^30^ ^]^ A gamut of advanced experimental techniques such as plasma spectroscopy,^[^ ^10^, ^11^ ^]^ shadowgraph imaging,^[^ ^9^, ^28^ ^]^ pump‐probe reflectivity,^[^ ^8^, ^31^ ^]^ X‐ray scattering^[^ ^32^, ^33^ ^]^ and X‐ray absorption spectroscopy^[^ ^33^, ^34^ ^]^ have made possible to deepen the understanding of NPs formation dynamics and the thermodynamic conditions during LAL, consistent with large‐scale molecular dynamics (MD) calculations.^[^ ^6^, ^7^ ^]^ For instance, these calculations showed the appearance of particle nuclei already in the early stage of plasma expansion in the cavitation bubble,^[^ ^6^, ^7^, ^11^, ^32^, ^35^ ^]^ and indicated that the nucleation and growth stages may last until several µs after the ablation.^[^ ^30^, ^33^, ^36^ ^]^ Size‐quenching effects in the presence of stabilizing compounds have been measured during and after the collapse of the cavitation bubble, providing a time‐resolved demonstration of the chemical reactions occurring throughout the process.^[^ ^32^ ^]^ Also, the chemical transformation of transition metals (Zn) during LAL in water and aqueous solutions of reducing and oxidizing species was probed by X‐ray absorption spectroscopy with µs time resolution, pointing to the critical role of the liquid phase in the process.^[^ ^33^, ^34^ ^]^

Noteworthy, the decomposition of the liquid solution, with the generation of reactive oxygen species (ROS), H_2,_ as well as organic radicals,^[^ ^37^, ^38^, ^39^, ^40^, ^41^ ^]^ offer unparalleled opportunities for driving the LAL‐produced colloids toward the desired composition and morphology.^[^ ^4^ ^]^ However, the liquid solution also influences the dynamics of the shockwave and the cavitation bubble (speed, pressure, lifetime, maximum size),^[^ ^5^, ^28^, ^42^ ^]^ complicating the disentanglement of the various contributions toward the final goal. Acting in an informed way on these processes is, thus, of utmost importance to improve the control of the LAL products, while also freeing its full potential for access to unconventional and non‐equilibrium nanostructures. Nevertheless, the literature so far has focused principally on single‐element compounds, leaving underexplored the time‐resolved dynamics of LAL applied at the formation of multimetallic and non‐equilibrium phases.

Here, the LAL of a multielement target is investigated aiming to improve the control over the local atomic structure of metastable nanophases. To this end, the effect of gas‐evolving solutes on the LAL of nonequilibrium nanoalloys is studied through an exhaustive approach for connecting the nanometric structure and composition of the laser‐generated nanophases with the reactivity of the environment where they are generated, as well as with the early (10–200 ns) and medium (1–100 µs) stage dynamics of the laser ablation process in the different environments. The LAL was performed with ns pulses in pure water or ethanol and in their solutions with additives developing gaseous species with different chemical reactivity such as O_2_, N_2_, and H_2_ (see Section S2, Supporting Information). The relevant instance of Au–Fe NPs has been selected because these nanostructures are of interest for multiple applications,^[^ ^12^, ^23^, ^24^, ^25^, ^43^, ^44^ ^]^ while also representing an ideal case study for the different chemistry of iron and gold and their temperature‐dependent miscibility.^[^ ^12^, ^21^, ^23^, ^24^, ^25^, ^26^, ^45^, ^46^ ^]^ It is well known that the Au–Fe nanoalloys obtained by LAL with ns pulses have less iron than in the initial alloy target and that multiple morphologies have been observed depending on the liquid environment, ranging from homogeneous alloys, iron‐gold core‐shells, gold‐iron oxide core‐shells, or metal‐oxide heterostructures.^[^ ^12^, ^26^, ^27^ ^]^ However, the relation between solutes and the nanoalloy structure at the nanometric scale, also defined as ultrastructure,^[^ ^14^, ^21^, ^22^ ^]^ has rarely been considered, despite the dramatic effects on the optical, magnetic, catalytic, and biocompatibility properties of these nanoalloys.^[^ ^12^, ^14^, ^21^, ^22^, ^24^, ^25^ ^]^ The use of shadowgraph imaging to resolve the laser‐induced shockwave and cavitation bubble dynamics in the different liquid environments is indispensable to complete the viewpoint of the effects introduced by gas‐evolving species.^[^ ^5^, ^28^, ^29^, ^42^ ^]^ In fact, it remains challenging to perform accurate molecular dynamics simulations on the interaction of ns laser pulses with solid matter in a liquid environment, and the attempts in this direction have considered only monometallic materials or ultrashort pulses, with limited information on the chemical processes occurring in the real experiments.^[^ ^5^, ^6^, ^7^, ^47^, ^48^ ^]^

Overall, this study provides a picture of the unexpected effects of solutes on the synthesis of nonequilibrium nanoalloys by LAL, with high resolution in terms of both the local structure of the Au–Fe NPs and the time evolution of the laser ablation process, ultimately facilitating future developments in the application of LAL for the achievement of innovative nanostructures with disruptive properties.

## 2 Results

### 2.1 Structural and Morphological Properties of Au–Fe Nanoalloys

**Figure 1:**
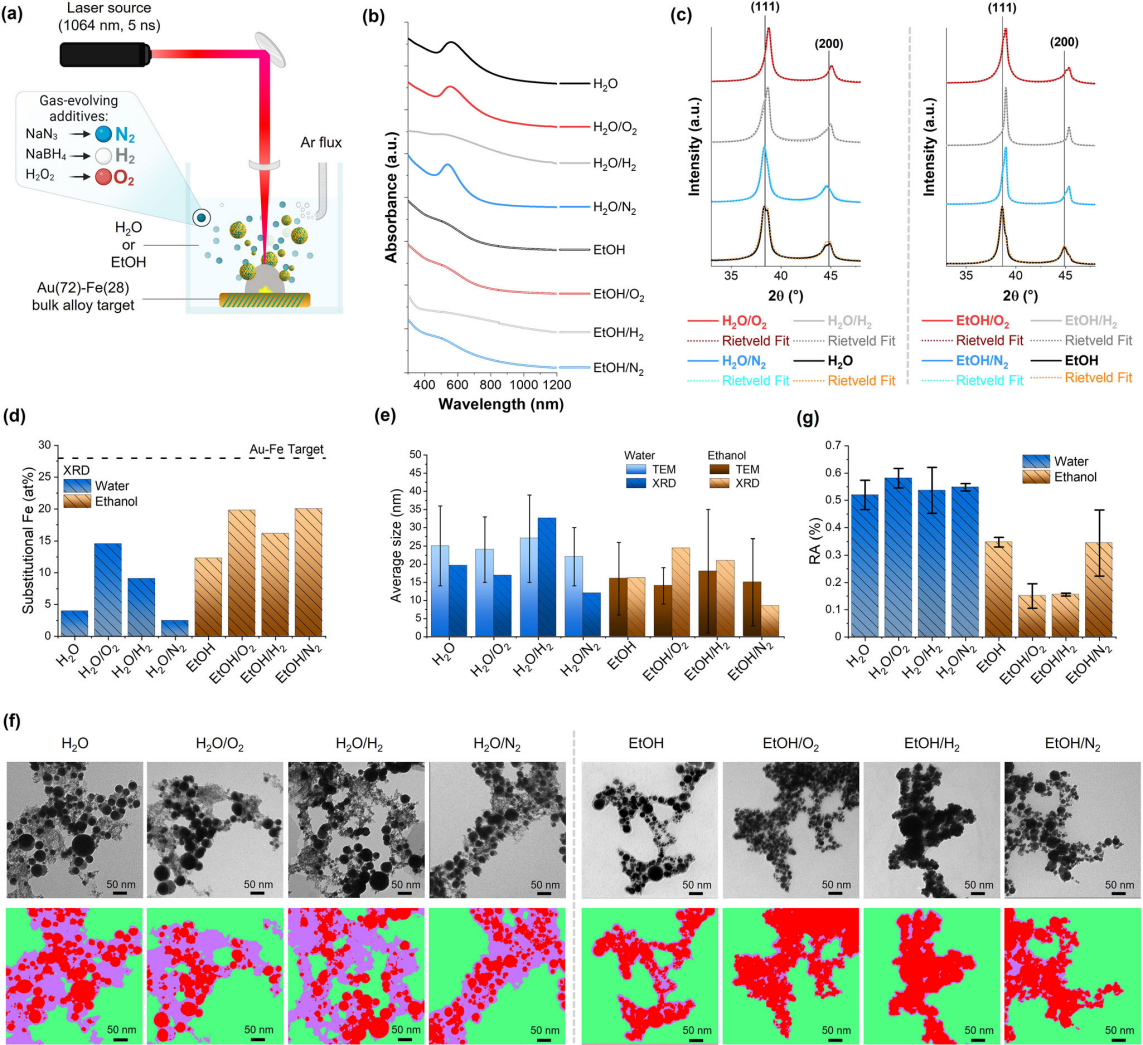
a) Sketch of the LAL synthesis process performed on an Au–Fe target in water or ethanol using gas‐evolving additives. Created with BioRender.com. b) UV–vis absorption spectra of the final dispersions in water of the eight samples obtained by LAL in water or ethanol. All spectra were normalized at 300 nm and vertically stacked for clarity. c) XRD and Rietveld refinement of the eight samples of Au–Fe NPs (see Figure S2, Supporting Information for full patterns). The diffractograms were normalized to the most intense reflection of the FCC pattern and vertically shifted for a clearer comparison. d) Iron at% is estimated from the contraction of the lattice parameter according to the Vegard law. e) The average size of NPs obtained from TEM images and crystallite size obtained from XRD through the Scherrer equation. f) Representative TEM images of the eight samples and corresponding segmentation based on hypodense regions (violet), hyperdense regions (red), and background (green). g) Average RA (%) of hypodense versus hyperdense regions for the eight samples, indicating the formation of synthesis byproducts in water with higher abundance than in ethanol.

The experiments considered the LAL of an Au–Fe (72–28 at%) alloy target in two solvents selected due to their different reactivity with laser‐ablated iron, which are H_2_O (oxidation of iron) and ethanol (limited oxidation of iron, iron carbides observed for LAL of pure iron targets but never observed for Au–Fe alloy targets).^[^ ^4^, ^5^, ^38^, ^49^ ^]^ The solvents were used pure or after the addition of NaBH_4_, NaN_3,_ and H_2_O_2_ (see summary in Table S1, Supporting Information). These gas‐evolving additives were selected for the release of, respectively, H_2_, N_2,_ and O_2_ during LAL. The LAL was performed under inert gas bubbling with a static cell at a low repetition rate (50 Hz) to reproduce the “ideal” single‐pulse laser‐ablation conditions, while also granting the productivity required for the following investigations (**Figure** **1**a). The products of LAL in the eight solutions were characterized according to a panel of structural techniques starting from the ensemble analysis and then moving to the single NP assessment until analytical measurements with nanometric resolution.

First, the optical properties of the Au–Fe nanoalloys were recorded, since they have a composition‐dependent localized surface plasmon (LSP) absorption band, with the highest intensity corresponding to pure gold NPs and damping when iron is included in the gold lattice as a substitutional element. LSP damping primarily results from the low‐frequency interband transitions introduced by iron *d*‐levels in the proximity of the Fermi energy of the alloy.^[^ ^14^, ^43^ ^]^ In the eight LAL‐generated samples, a gold‐like LSP is identified in the proximity of 540–550 nm for the H_2_O, H_2_O/N_2,_ and H_2_O/O_2_ environments (Figure 1b). In the EtOH, EtOH/N_2_ and EtOH/O_2_ samples, the LSP is almost completely quenched, providing strong evidence of alloying. The spectra of the samples obtained in H_2_O/H_2_ and EtOH/H_2_ are broad and feature a high optical extinction in the near‐infrared, both indicative of plasmon hybridization due to strong coupling between the metal NPs, preventing the evaluation of NPs composition. Note that all samples were collected and washed by centrifugation and redispersed in pure water to avoid any influence on the optical properties due to the different solvents or solutes.

To properly identify the crystalline phases in the samples, powder X‐ray diffraction (XRD) and Rietveld refinement were performed. All the diffractograms (Figure 1c; Figure S2, Supporting Information) exhibit only the reflections of the face‐centered cubic (FCC) phase typical of noble metals like Au, i.e., no peaks proper of iron oxides or iron carbides are found. Note that the reflections of α‐Fe overlap with those of the FCC Au,^[^ ^23^ ^]^ making the detection of metallic iron from XRD impossible. Also, crystalline iron oxide below some wt% cannot be detected beneath the reflections of a high Z compound such as Au. However, the lattice parameters in several samples are significantly smaller than in pure gold, which is the effect of substitutional Fe atoms replacing the larger Au atoms in the FCC cell (see Table S2, Supporting Information for details).^[^ ^12^, ^14^, ^43^ ^]^ The lattice parameters change with the LAL environment, corresponding to different alloy compositions based on the Vegard law^[^ ^12^, ^14^, ^43^ ^]^ (Figure 1d). More specifically, the average content of substitutional iron is only 4 at% for the H_2_O and 12 at% for the EtOH samples, versus 28 at% in the target. The Fe at% increases to 20 at% in EtOH/N_2_, 16 at% in EtOH/H_2,_ and 20 at% in EtOH/O_2_, indicating that gas evolution opposes dealloying in ethanol. This is confirmed even in water, where the substitutional iron content reaches 9 at% in H_2_O/H_2_ and 14.5 at% in H_2_O/O_2_, which is unexpected considering the oxidizing effect of H_2_O_2_. In H_2_O/N_2_, instead, the substitutional iron fraction is 2 at%, comparable to the H_2_O sample. This is attributed to the stability of N_3_^−^ in water^[^ ^50^ ^]^ and the consequently low gas evolution during LAL compared to the other additives.

**Figure 2:**
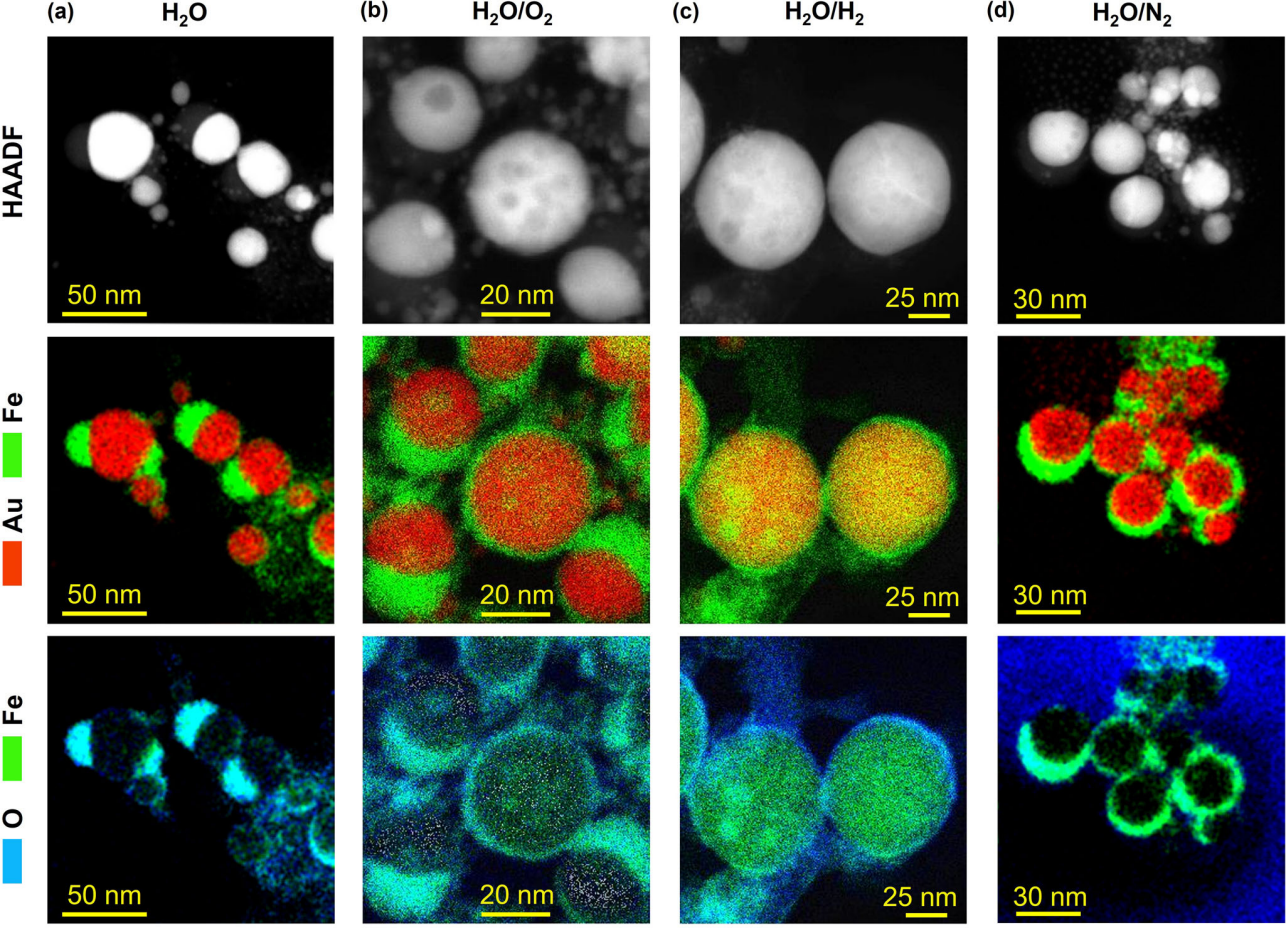
STEM‐HAADF (top), EDX map of Au M and Fe K lines (middle), and EDX map of Fe and O K lines (bottom) of representative NPs from the H_2_O a), H_2_O/O_2_ b), H_2_O/H_2_ c) and H_2_O/N_2_ d) samples.

In most samples, the Rietveld refinement identified two populations of nanoalloys with different lattice parameters and crystallite sizes (according to the Scherrer formula, Table S2, Supporting Information). In these cases, the smaller crystallites are always associated with a lower Fe content. Hence, more information on the NP's size and morphology was obtained by transmission electron microscopy (TEM). The average Feret size measured from the TEM images (Figure 1e) is comparable within the same solvent (22–27 nm for water and 14–16 nm for ethanol). The size histograms (Figure S3, Supporting Information) all have a lognormal distribution with a peak at small sizes and a tail due to larger NPs, consistent with the LAL using ns pulses.^[^ ^4^, ^5^, ^6^ ^]^ Therefore, the gas‐evolving solutes have no distinctive effects on the size distribution of the nanoalloys, which appears related only to the solvent. Furthermore, relevant morphological differences are associated with the solvent, due to the presence of a matrix with low electronic contrast (i.e., low density) surrounding the spherical metal NPs obtained in water (Figure 1f; Figure S4, Supporting Information). A machine‐learning model was applied for the segmentation of TEM images into the hyperdense (metal phase) and hypodense (metal oxides or hydroxides, ultrafine NPs, carbon‐based residuals) regions of the samples. Although limited to the bi‐dimensional projection of the nanostructures recorded in TEM images, this approach allowed a quantitative evaluation of the relative abundance (RA, in %) of the hypodense region versus the total surface of the nanostructures (hypodense + hyperdense). The RA can be compared with the composition of the nanoalloys to gather insights about the fate of the iron lost during LAL (Figure 1g). In water samples, the RA was equally high in samples exhibiting dealloying (H_2_O and H_2_O/N_2_) as well as in those featuring a higher abundance of substitutional Fe (H_2_O/O_2_ and H_2_O/H_2_). Indeed, crescents and shells around metal nanospheres, as well as distinctive Janus morphologies, are ubiquitous in the TEM images of H_2_O, H_2_O/N_2,_ and H_2_O/O_2_ samples, with the sole exception of the H_2_O/H_2_ sample (Figure 1f; Figure S3, Supporting Information). These heterostructures are made of iron oxide, as confirmed by energy dispersive X‐ray spectroscopy (EDX) elemental maps and linescans (**Figure** **2**; Figure S5, Supporting Information) of the Au L‐line, Fe K‐line and O K‐line in H_2_O and H_2_O/N_2_ samples. High‐resolution TEM (HRTEM) and the related fast Fourier transform (FFT) patterns of the Janus NPs confirmed the identification of the interplanar distance of 0.265 ± 0.005 nm, matching magnetite Fe_3_O_4_, in the low contrast crescent region, and of 0.240 ± 0.005 nm, matching FCC Au, in the high contrast NP region (Figures S6 and S7, Supporting Information). The presence of crescents, core–shells, and Janus NPs with sharp metal/oxide interfaces are all indicative of dealloying and oxidation of the iron atoms initially present within an original bimetallic Au–Fe nanophase at high temperature, when the atomic mobility is still large. This homogeneous Au–Fe nanophase is necessarily generated during the first stage of the LAL while undergoing different structural transformations depending on the liquid environment.

Despite the presence of nanocrescents in H_2_O/O_2_ and the large RA in the H_2_O/O_2_ and H_2_O/H_2_, both samples prevalently contain Au–Fe nanoalloys based on the lattice parameter contraction. Hence, EDX elemental mapping with nanometric resolution was performed to further substantiate the XRD result. In the H_2_O/O_2_ sample (Figure 2b; Figure S8, Supporting Information), EDX mapping confirmed the coexistence of iron and gold in the metallic phase, with a composition (Fe 18 at%) close to that estimated from XRD (14.5 at%). Furthermore, iron oxide clusters are observed inside the NPs (see STEM‐HAADF and the Fe/O EDX maps in Figure 2b; Figure S8, Supporting Information). Overall, dealloying in the H_2_O/O_2_ sample was incomplete, contrary to the H_2_O and H_2_O/N_2_, resulting in a peculiar ultrastructure with a mixture of oxidized iron embedded in a bimetallic Au–Fe matrix, surrounded by iron oxide crescents and shells.

In the NPs of the H_2_O/H_2_ sample, the STEM‐HAADF and EDX mapping identified a thin shell of iron oxide and iron‐rich clusters within the Au–Fe matrix (Figure 2c and Figure S8, Supporting Information). Furthermore, the average Fe content in the analyzed NPs resulted in as high as 36 at% (Figure S8, Supporting Information). Apart from the experimental error, this value is well above the 9.0 at% of substitutional Fe evaluated from the contraction of the lattice parameter in the XRD pattern (Figure 1d), as well as the 18 at% detected by EDX on the H_2_O/O_2_ sample. The oxygen signal is observed in concomitance of the iron clusters, indicating a possible iron oxide nature also in these cases (Figure S8, Supporting Information). Sample H_2_O/H_2_ has higher iron content from EDX at the single particle level, but lower substitutional iron from XRD compared to sample H_2_O/O_2_, which also shows clear indications of iron oxidation. This discrepancy suggests the potential precipitation of metallic iron in the NPs of sample H_2_O/H_2_.

When considering the four samples in ethanol, the morphology of NPs is always spherical and with a homogeneous contrast, with a limited presence of the hypodense component, as expected for regular nanoalloys (Figure 1f; Figures S3 and S4, Supporting Information). The EtOH sample has the largest RA among the samples in ethanol, coinciding with the lowest at% of substitutional iron according to the XRD analysis. In the EtOH/N_2_ sample, the RA is also large but associated with a wide standard deviation, because the hypodense region appears with lower density and is quantitatively less abundant. However, this may be also attributed to carbonaceous compounds. The RA is low in the EtOH/O_2_ sample, whose NPs appear morphologically equivalent to the previous two samples. Importantly, there is no evidence of iron oxide crescents or shells. The EtOH/H_2_ sample has the lowest RA and exhibits a dendritic morphology, i.e., without distinct interfaces which are favored by the formation of oxidized iron layers between the metal NPs (see also Figure S3, Supporting Information). Overall, based on the morphological analysis of water and ethanol samples, the H_2_ environment resulted in the most effective in limiting the oxidation of iron during the formation of nanoalloys by LAL. Besides, no stabilizers or other chemical compounds were added, therefore, the plasmon coupling of metal NPs is possible in the EtOH/H_2_ samples without the existence of an iron oxide interface.

**Figure 3:**
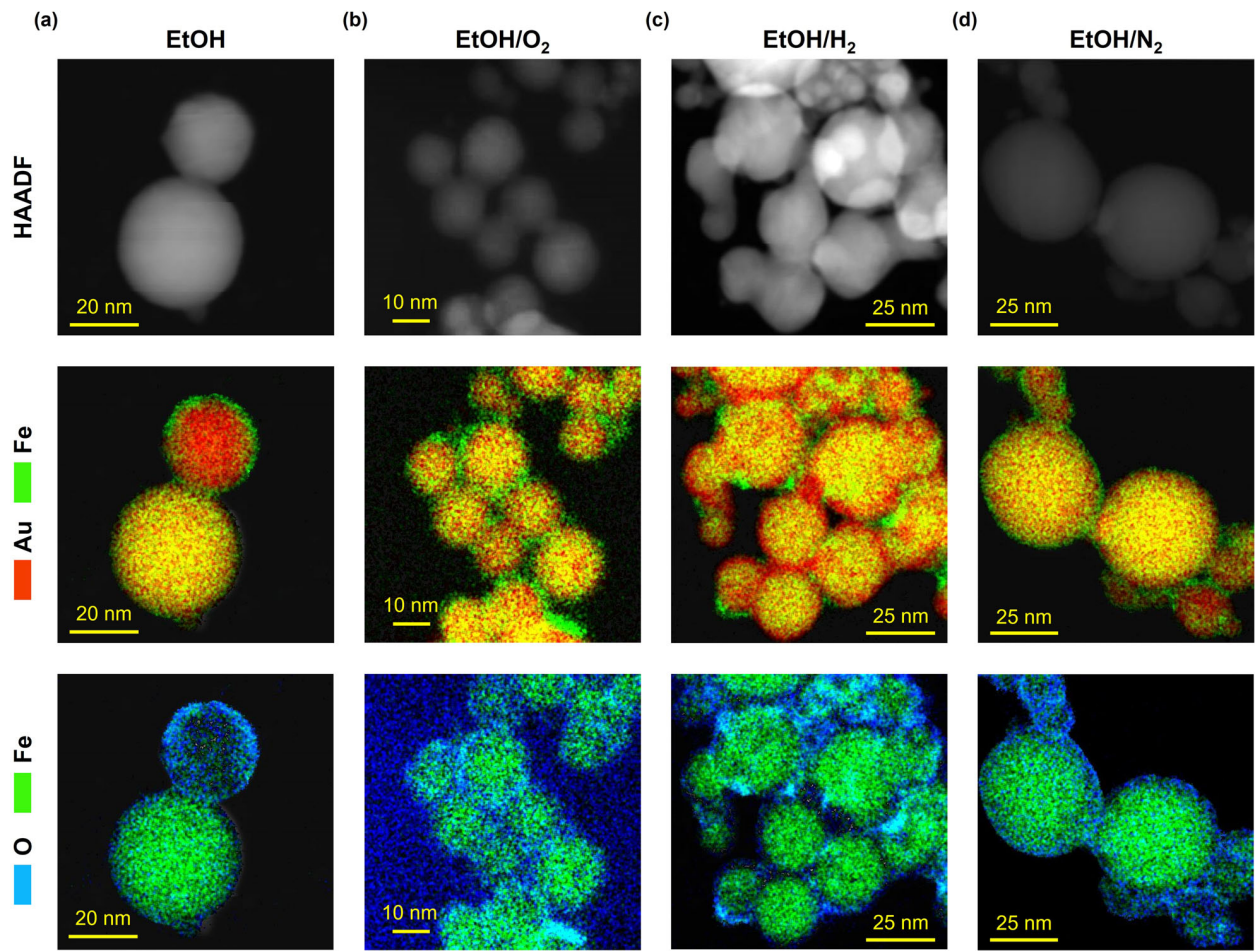
STEM‐HAADF (top), EDX map of Au M and Fe K lines (middle), and EDX map of Fe and O K lines (bottom) of representative NPs from the EtOH a), EtOH/O_2_ b), EtOH/H_2_ c) and EtOH/N_2_ d) samples.

To properly identify the diverse effects of gas‐evolving additives on the ultrastructure of the NPs, the EDX elemental mapping with nanometric resolution was indispensable (**Figure** **3**). The NPs in the EtOH sample have a homogeneous Au–Fe core surrounded by a nanometric shell of oxidized iron (Figure 3a and additional STEM‐EDX maps in Figure S9, Supporting Information). In the EtOH/N_2_ and EtOH/O_2_ samples, iron oxide is also observed at particle surface, predominantly as nano‐crescents rather than as a complete shell. The HRTEM and the corresponding FFT patterns confirmed the FCC crystalline structure of these NPs, as previously identified with XRD (Figures S10–S12, Supporting Information), while the reflections of magnetite are occasionally found at the surface. In the EtOH/H_2_ sample (Figure 3c; Figure S9, Supporting Information), the EDX maps revealed that NPs have the opposite surface morphology, namely a nanometric thin skin of Au surrounding a bimetallic Au–Fe core. Consequently, no magnetite reflections were identified in HRTEM images and the corresponding FFT patterns (Figure S13, Supporting Information). It is worth noting that, contrary to the H_2_O/H_2_ sample, the reducing H_2_ environment of the EtOH/H_2_ experiment was not associated with the precipitation of well‐defined iron clusters inside the bimetallic core. In general, iron‐rich clusters were not identified in the samples of the ethanol series. According to the EDX quantitative elemental analysis, the NPs among the four samples also have a similar average Fe content, namely 18 at% in EtOH (12 at% from XRD), 18 at% in EtOH/N_2_ (20 at% from XRD), 22 at% in EtOH/O_2_ (20 at% from XRD) and 20 at% in EtOH/H_2_ (16 at% from XRD). The EDX composition is close to the substitutional iron content obtained from XRD in the EtOH/N_2_ (20 at%), EtOH/O_2_ (20 at%), and EtOH/H_2_ (16 at%) samples, but larger than in the EtOH sample (12 at%), where the elemental mapping also indicated the signs of surface oxidation.

### 2.2 Cavitation Bubble Dynamics

**Figure 4:**
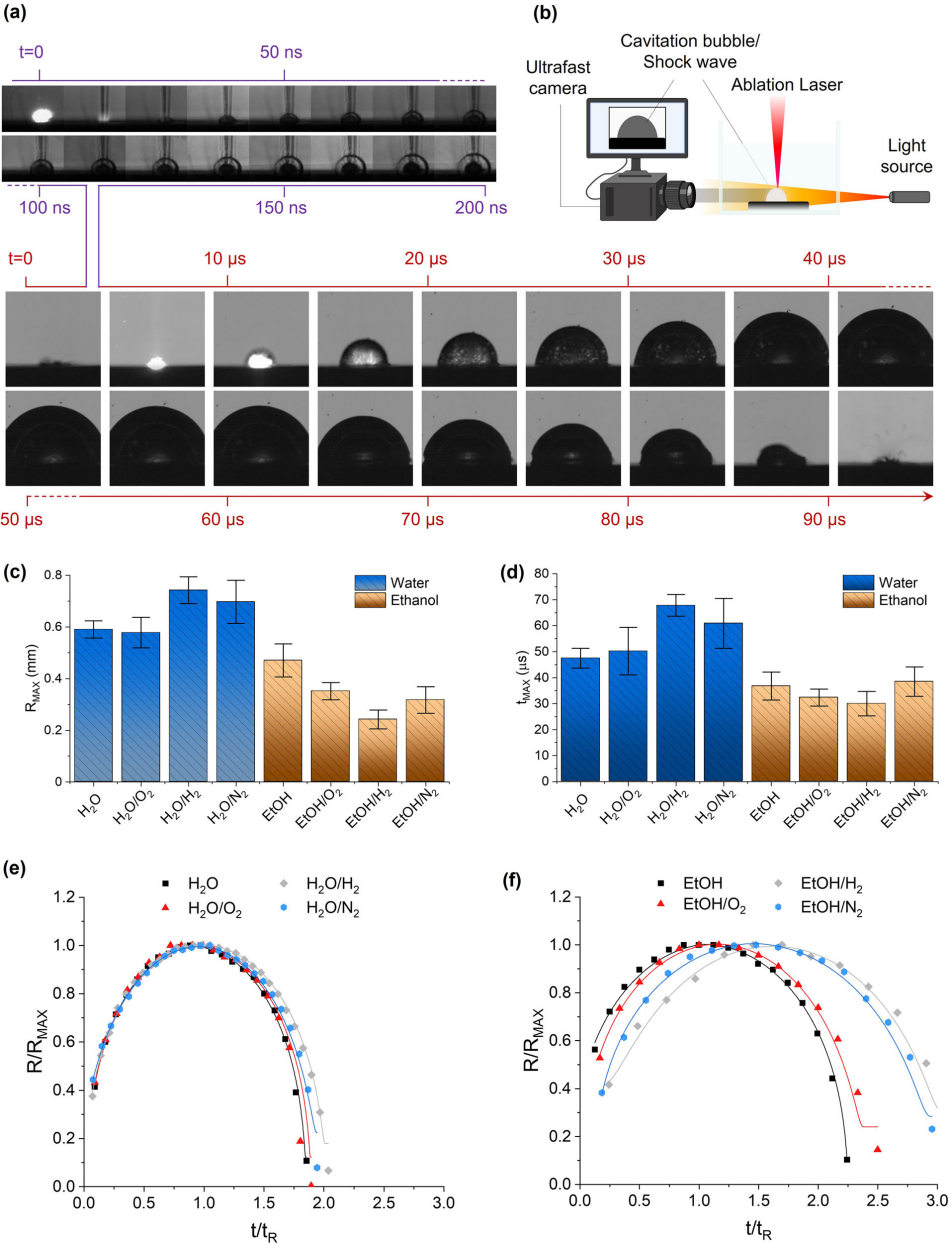
a) Lower panel: representative snapshots of shadowgraph imaging of the LAL‐induced cavitation bubble (tens of µs time scale) in the EtOH environment, recorded with the setup sketched in (b, created with BioRender.com). A pattern of ultrabright LEDs is used as the illumination source, allowing the registration of the bubble shadow with an ultrafast camera. As a comparison, the dynamics of the LAL‐induced shockwave, obtained with a multi‐channel framing camera and a pulsed diode laser light source is shown in the upper panel (ns time scale) for the same liquid environment. c,d) Plot of the maximum size (R_MAX_, c) reached from the bubbles and the associated lifetime (t_MAX_, d) for the eight samples in water and ethanol. The error bars correspond to the level of confidence at 95% on the average over 35 independent measurements. e,f) Plot of the cavitation bubble dynamics in water (e) and ethanol (f) and fit with the RP equation using the Euler method. The dynamics are reported in terms of the non‐dimensional values obtained by dividing t by the Rayleigh collapse time (t_R_) and the size (R) by the maximum radius (R_MAX_).

In the synthesis of NPs by LAL, the cavitation bubble acts as a microreactor that exists for the critical timespan comprised between tens of nanoseconds and hundreds of microseconds after absorption of the laser pulse.^[^ ^4^, ^28^, ^29^, ^32^, ^38^ ^]^ The interior of the cavitation bubble is predominantly composed (> 90%) of solution species.^[^ ^5^, ^29^ ^]^ Therefore, the effect of gas‐evolving solutes on the dynamics of the cavitation bubble has been investigated to verify its relationship with the structure of the nanoalloys (**Figure** **4**a,b). The optimal conditions were achieved using a fluxed cell to avoid the overlap of ablated material with the laser pulses and to remove the persistent microbubbles, which are especially abundant in the presence of gas‐evolving solutes. The NPs and the microbubbles may affect the reproducibility of the experiment by attenuating the energy delivered to the target.^[^ ^47^ ^]^ Furthermore, the persistent microbubbles altered the shape of the cavitation bubble in a non‐reproducible way, preventing any quantitative analysis. In the solution refreshed by the liquid flux, instead, it was possible to measure the radius of the bubbles as a function of time, and to describe their dynamics with finite element methods according to the Rayleigh–Plesset (RP) theory.^[^ ^28^, ^29^ ^]^ According to the statistics on a minimum of 35 cavitation bubbles for each sample, the main bubble parameters such as the maximum size (R_MAX_, Figure 4c) and the corresponding time (t_MAX_, Figure 4d) only differ between the water (0.58–0.74 mm, 47–68 µs) and ethanol (0.24–0.47 mm, 29–38 µs) groups, without a specific effect attributable to the gas‐evolving additives. In particular, the R_MAX_ and t_MAX_ in H_2_O/H_2_ (0.74 ± 0.05 mm, 68 ± 4 µs) and H_2_O/N_2_ (0.70 ± 0.08 mm, 61 ± 9 µs) are larger than in H_2_O/O_2_ (0.58 ± 0.06 mm, 50 ± 9 µs) and H_2_O (0.59 ± 0.03 mm, 47 ± 4 µs), whereas the opposite is observed in ethanol where the samples with gas evolving additives (EtOH/N_2_, EtOH/H_2_, EtOH/O_2_) have lower R_MAX_ and t_MAX_ (respectively, 0.32 ± 0.05 mm and 38 ± 6 µs, 0.24 ± 0.04 mm and 30 ± 5 µs, 0.35 ± 0.03 mm and 32 ± 4 µs) than in EtOH (0.47 ± 0.06 mm, 37 ± 5 µs). Overall, the R_MAX_ and t_MAX_ values result in the absence of any trend or correlation with the fraction of substitutional iron in the FCC Au–Fe nanoalloys obtained from the Vegard law (see the plots in Figure S14, Supporting Information).

Concerning the bubble dynamics, its agreement with the RP theory is best appreciated by resorting to the reduced quantities R/R_MAX_ and t/t_R_ (Figure 4e,f),^[^ ^28^, ^29^, ^51^ ^]^ where t_R_ is the Rayleigh collapse time (see Methods in Supporting Information for further details). The dynamics in the water samples are all well‐fitted with the RP model, exhibiting symmetric expansion and collapse stages versus t/t_R_. In ethanol, the RP model also fitted the experimental data, although the dynamics is asymmetric because of a slower collapse stage in the samples with gas‐evolving additives. According to the RP model, the internal bubble pressure at t_MAX_ (p_MAX_) can be evaluated in all samples (Figure S15, Supporting Information), without any appreciable dependence on the gas‐evolving solutes.

### 2.3 Shockwave Dynamics

Motivated by the absence of a unique effect of the gas‐evolving solutes on the dynamics of the cavitation bubble, the early stage of LAL was investigated by shadowgraphy with a multi‐channel framing camera capturing one image every 13 ns (see Methods in Supporting Information for details). The experiment was carried out in H_2_O and EtOH as a reference, and in the H_2_O/O_2_ and EtOH/O_2_ environments, because the nanoalloys were achieved with a higher iron content than in the pure solvents despite the presence of an oxidizing additive as H_2_O_2_. This must be indicative of a physical‐chemical alteration of LAL conditions that goes beyond the change in the chemical environment. Such a critical aspect became evident using an oxidizing additive (H_2_O_2_) to complement the observations with reducing additives (NaBH_4_ and NaN_3_). Again, the experimental conditions of shadowgraph imaging were set to properly record the early dynamics in reproducible conditions and without interference from persistent microbubbles, while keeping comparable laser pulse fluence and ns pulse duration as in the previous experiments.

**Figure 5:**
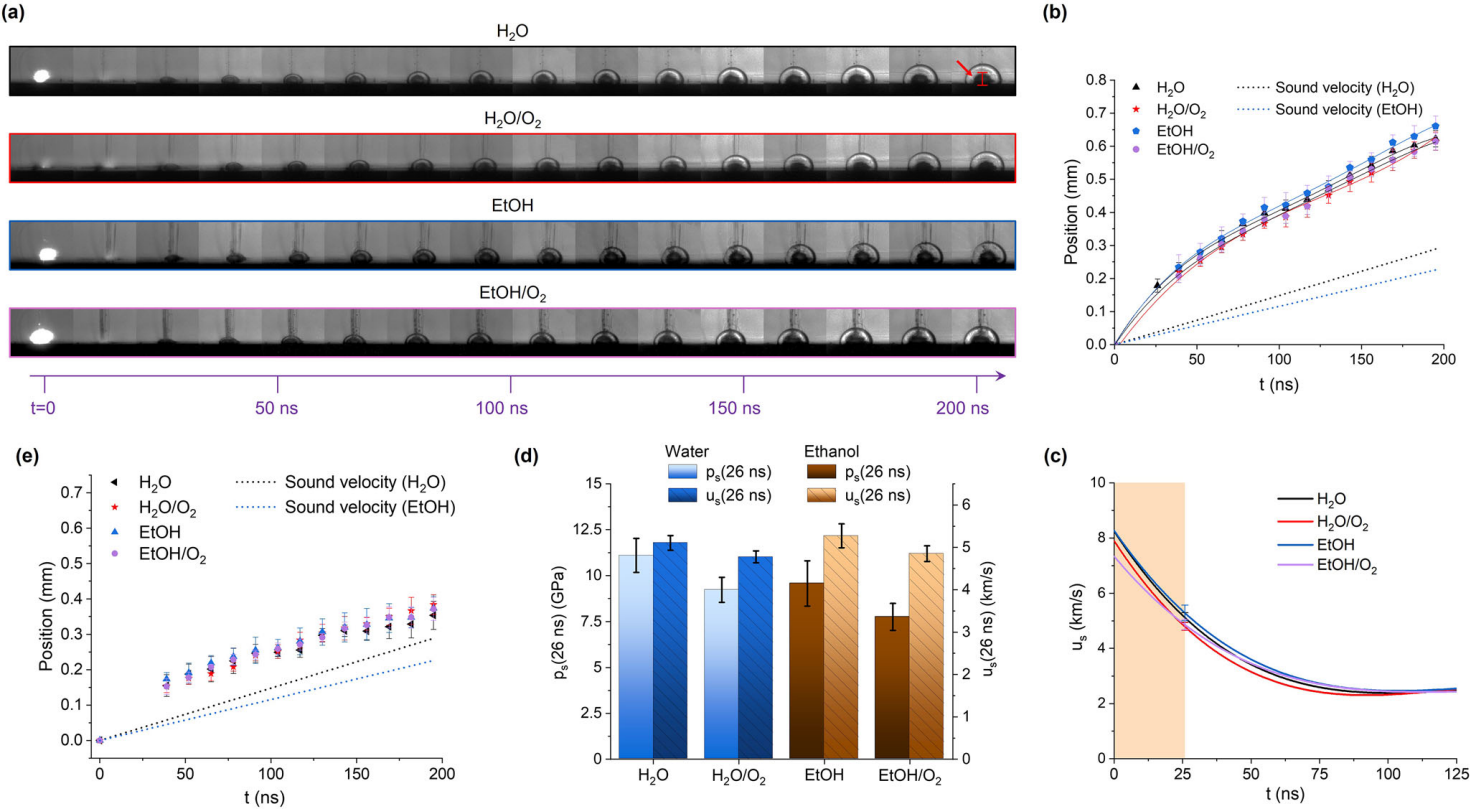
a) Representative shadowgraph images of the shockwave generated by LAL on the Au–Fe target in the H_2_O, H_2_O/O_2_, EtOH, and EtOH/O_2_ samples. The red arrow indicates the early rise of the cavitation bubble. b) Dynamics of the shockwaves in the four environments considered. The supersonic speed is evidenced by the comparison with the sound speed in water (dotted black line) and ethanol (dotted blue line). c) Plot of the shock wave velocity (u_s_) in the 0–125 ns interval. The orange‐shaded area corresponds to the region in which direct experimental data are not available. d) Plot of the shock front u_s_ and pressure (p_s_) calculated at 26 ns (the first experimental timepoint available), indicating lower values for H_2_O/O_2_ and EtOH/O_2_ than in the corresponding pure solvents H_2_O and EtOH. e) Early dynamics of the cavitation bubble in the four environments considered. For tens of ns after 39 ± 5 ns, the expansion occurs at sound speed, as evidenced by the comparison with the sound speed in water (dotted black line) and ethanol (dotted blue line).

With nanosecond time resolution, the shockwave dynamics can now be recorded (**Figure** **5**a), evidencing a supersonic velocity of the early released shockwave in both solvents (Figure 5b), and in agreement with the literature.^[^ ^5^, ^28^, ^31^, ^51^ ^]^ Indeed, within the error over five repeated experiments, the shockwave dynamics are comparable in all the tested environments, with a difference resulting sensibly lower than between the sound speeds of the two liquids. The presence of the O_2_‐evolving additive did not alter these dynamics. The first derivative of the shockwave position versus time, fitted with a fourth‐order polynomial, provides the evolution of propagation velocity (u_s_) in the four environments (Figure 5c). The velocity is maximum in the first ns after the absorption of the laser pulse, decreasing toward the sound speed after a hundred ns, in all cases. Note that the shockwave is clearly appreciable above the target only 26 ± 5 ns after the laser pulse, therefore the velocities at lower times (orange‐shaded area in Figure 5c) result from the interpolation of the polynomial fit in the 0–26 ns range. The u_s_ at t = 0 ns are in a range between 7.3 and 8.3 km ^−1^s but a more reliable comparison is given at 26 ns, where the first experimental points are recorded. Here, velocities are 5.1 ± 0.2 and 4.8 ± 0.1 km ^−1^s in, respectively, H_2_O and H_2_O/O_2_, and 5.3 ± 0.3 and 4.8 ± 0.2 km ^−1^s in, respectively, EtOH and EtOH/H_2_O_2_, with a decrease of 0.4–0.5 km ^−1^s in the presence of the O_2_‐evolving additive.

The pressure at the shock front (p_s_) is another parameter defining the first stages of LAL, which is linked to u_s_ through the Hugoniot curve for liquids^[^ ^52^, ^53^ ^]^ (see Methods in S.I. for details). At t = 0 ns, ps is the same (31 GPa) in H_2_O and H_2_O/O_2_, but lower in EtOH (27 GPa) and EtOH/O_2_ (21 GPa). At t = 26 ns (more reliable), the trend of p_s_ follows that of lower u_s_ in the presence of oxygen‐evolving additives (Figure 5d), resulting 11.1 ± 0.9 GPa in H_2_O, 9.2 ± 0.7 GPa in H_2_O/O_2_, 9.6 ± 0.2 GPa in EtOH and 7.8 ± 0.7 GPa in EtOH/O_2_.

The ns time resolution also allowed monitoring the early rise of the cavitation bubble (red arrow in Figure 5a), indicating its appearance already after 39 ± 5 ns for all samples. The bubble dynamics is slower than the shockwave and equivalent to the sound speed for tens of ns after 39 ± 5 ns (Figure 5e). The trend of the shock wave and bubble velocity was consistent with the previous results in water.^[^ ^51^ ^]^ The rapid bubble appearance is indicative of its formation by a cavitation phenomenon,^[^ ^5^, ^28^ ^]^ i.e., by a negative pressure peak following the shock propagation,^[^ ^54^, ^55^ ^]^ instead of heat transfer from the plasma to the liquid. This is reasonable since the pressure of the shock front reaches a range of values between 7 and 12 GPa at 26 ns, as previously observed.^[^ ^28^ ^]^

## 3 Discussion

### 3.1 Atomic Structure

**Figure 6:**
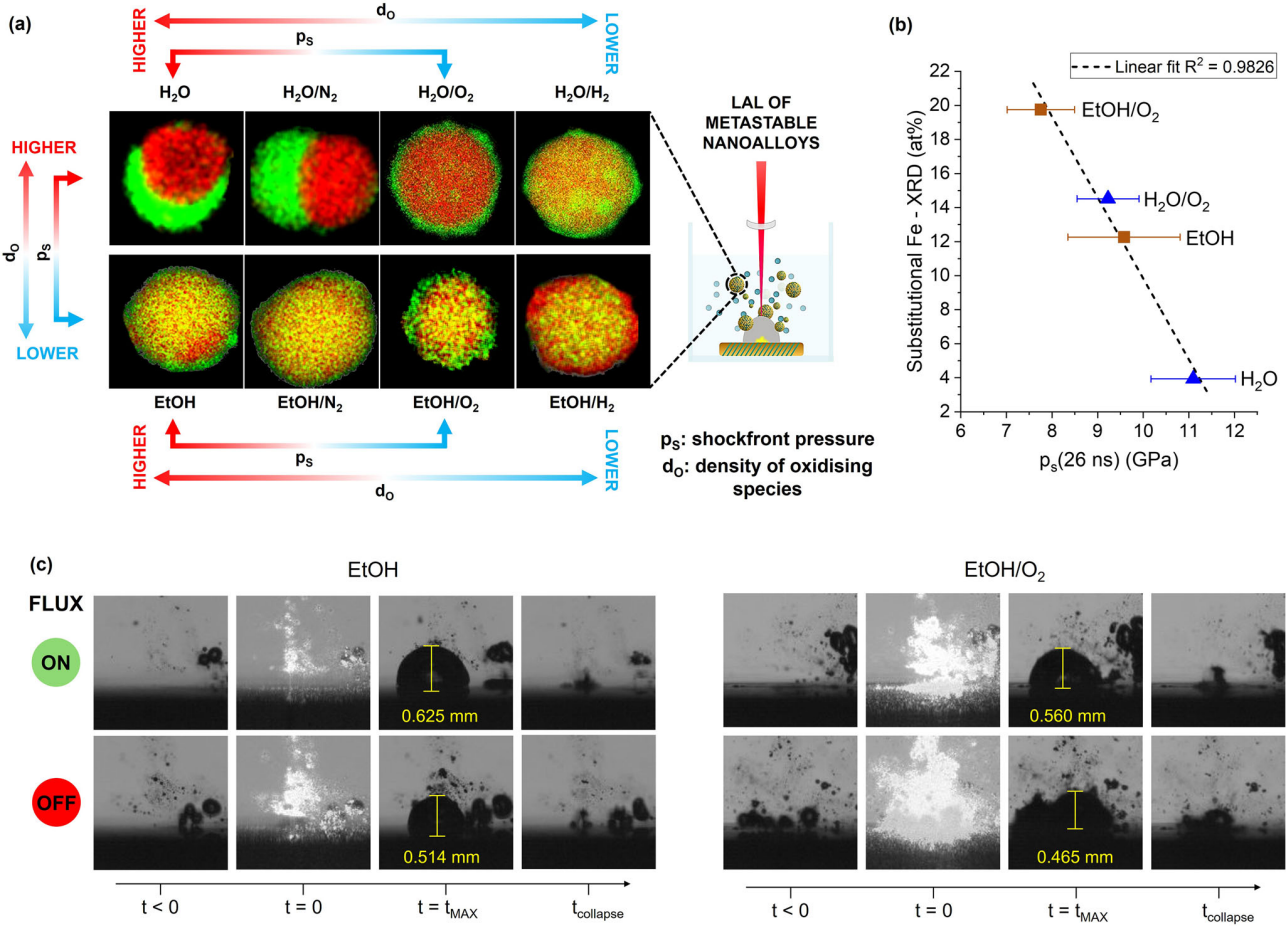
a) Summary of the interplay between laser pulses and liquid environment for molding the ultrastructure of metastable Au–Fe nanoalloys: moving from a higher density of oxidative species (d_O_), as in H_2_O, to a lower d_O_, as in EtOH, and moving from a higher to lower shockfront pressure (p_s_), again as from H_2_O to EtOH, the formation of the nanoalloys is favored. Besides, different ultrastructures correspond to the different combinations of oxidative environment and p_s_. The EDX images refer to the most representative structures observed in each sample. b) Plot of the substitutional Fe at% (from XRD) versus the shock front pressure (p_s_) at 26 ns in the H_2_O, H_2_O/O_2_, EtOH, and EtOH/O_2_ samples, showing a linear correlation with an R^2^ of 0.9826. Error bars on p_s_ are obtained by error propagation (for details see Statistical analysis paragraph in S.I.); errors on Fe at% are reported in Table S2 (Supporting Information) c) Shadowgraph images collected in EOH and EtOH/O_2_ environments with (top) and without (bottom) fluxing the liquid at each laser pulse. The persistent microbubbles interfere with the cavitation bubble, leading to a smaller radius. The persistent microbubbles are more abundant in the liquid with the gas‐evolving solute.

The realization of metastable nanoalloys with controlled composition and finely tuned ultrastructure is an ambitious and challenging goal in nanotechnology. Here it is shown that this goal can be met through LAL of bulk targets made of pure metals and immersed in the most used green liquids (water and ethanol) containing a low concentration (0.01 wt%) of common low‐cost gas‐evolving solutes. By changing the combination of liquids and solutes, Au–Fe nanoalloys with a variety of ultrastructures were achieved (**Figure** **6**a), such as Janus NPs made of an iron‐poor Au–Fe alloy coupled with iron oxide (H_2_O, H_2_O/N_2_), Janus NPs made of the Au–Fe alloy coupled with iron oxide and containing clusters of iron oxide (H_2_O/O_2_), Au–Fe alloys containing iron‐rich clusters (H_2_O/H_2_), homogeneous Au–Fe alloys with a shell of oxidized iron (EtOH, EtOH/N_2_, EtOH/O_2_), and dendritic Au–Fe alloys with a skin of Au (EtOH/H_2_). The room temperature Au–Fe phase diagram does not include thermodynamically stable alloys beyond a few at% of one metal in the other.^[^ ^12^, ^21^, ^23^, ^24^, ^43^ ^]^ Nonetheless, the Au *d*‐electrons can hybridize with the *d*‐electrons of Fe,^[^ ^14^, ^43^ ^]^ allowing for local thermodynamic minima where the metastable alloy can exist. For these nanoalloys, a wide range of nanotechnological applications has been reported or foreseen. These applications range among theranostics, optical labeling, optical spectroscopy, sensing, catalysis, and magneto‐optics, by leveraging the peculiarities of each specific morphology and composition of the Au–Fe NPs (see Table S3, Supporting Information for a summary for each specific type of NPs morphology).

Yet, in terms of control on nanoalloy stoichiometry and a variety of atomic structures, the results of this study expanded what achieved so far starting from bimetallic bulk targets of immiscible noble metals and transition metals.^[^ ^12^, ^23^, ^26^, ^43^ ^]^ In the previous studies (see Table S4, Supporting Information for a summary of previous laser‐assisted synthesis of Au–Fe NPs), the most complex and interesting ultrastructures required the use of different laser sources, target stoichiometry, target structure, or liquids with lower versatility than water and ethanol.^[^ ^21^, ^22^, ^23^, ^24^, ^25^, ^27^ ^]^

According to the calculations of Gibbs free energy in bimetallic NPs with positive mixing enthalpy (see Section S4 in S.I.), a size threshold exists in Au–Fe NPs for the phase separation into core‐shell structures, with the shell composed of the metal with the lower interface energy (Au).^[^ ^21^, ^22^, ^23^, ^56^ ^]^ The thermodynamic advantage for the core‐shell Fe‐Au morphology increases with the size of the NPs and the iron fraction, namely, the size threshold for core‐shell formation is lower when the iron fraction in the alloy is larger.^[^ ^21^, ^23^ ^]^ The calculations reported in Figure S16 (Supporting Information) confirm the existence of a thermodynamic driving force for element segregation for most of the NPs analyzed with EDX mapping in this study. However, the NPs with a gold shell were identified only in the EtOH/H_2_ sample, whereas all the other samples contained NPs with an iron oxide shell (Figures 2 and 3 and Figures S5, S8, and S9, Supporting Information). In some cases (H_2_O/O_2_, H_2_O/H_2_), iron‐rich clusters were observed, which in literature have been identified as the intermediate stage of segregation into a core‐shell structure.^[^ ^21^, ^22^ ^]^ This is explained also by the higher melting temperature of iron (1811 K) compared to gold (1337 K).^[^ ^21^, ^22^ ^]^ In this study, the oxygen signal was found in the iron‐rich clusters by the EDX analysis. These results may be indicative that iron oxidation contributes to the interruption of gold segregation at the surface of NPs, with the formation of the core‐shell morphology.^[^ ^21^, ^22^, ^23^, ^56^ ^]^ The interface energy of iron oxide is much lower than that of metal Fe or Au and their alloys.^[^ ^57^ ^]^ In addition, the absorption of water and oxygen and the oxidation of Fe by the formation of, respectively, surface hydroxides and oxides is thermodynamically favored at any Au–Fe alloy composition.^[^ ^12^ ^]^ Therefore, in the presence of oxidizing species, the appearance of an iron oxide shell around the Au–Fe NPs will be always favored compared to the formation of an Au shell. The lowest surface oxidation of the Au–Fe NPs is observed in the EtOH/H_2_ sample, where also a thin Au shell is detected. Noteworthy, the iron content in the EtOH/H_2_ NPs analyzed by EDX is 20 at%, lower than the 30–50 at% of core‐shell NPs described in previous studies.^[^ ^21^, ^22^, ^23^, ^56^ ^]^

### 3.2 Time‐Resolved Experiments

Because of the immiscibility and different electrochemical reduction potentials of Fe and Au, the physical‐chemical mechanisms behind the formation of the Au–Fe NPs result from the intricate combination of specific chemical processes and modified thermodynamic paths made possible during LAL. This prompted the present dedicated experimental investigation, which was not performed before for bimetallic metastable compounds. In particular, the H_2_O/O_2_ sample contained nanoalloys richer in substitutional iron than the H_2_O sample without the oxidizing additive, and this contradicts the expectations based on the chemical contribution of the liquid (water) and the solute (H_2_O_2_) environment. A similar result was found in EtOH/O_2_, because of the relatively high amount of substitutional iron in the Au–Fe FCC lattice of the NPs compared to what was found in solution without the oxidizing additive as pure EtOH, EtOH/H_2,_ and EtOH/N_2_. The efficient formation of substitutional Au–Fe nanoalloys in the presence of H_2_O_2_ can be explained only by the change in the dynamics of the process, and not just with a purely chemical phenomenon. This provided another motivation for the investigation of the cavitation bubble and the evolution of the shockwave with time‐resolved experiments at the µs and ns timescale. Previous time‐resolved investigations on LAL in the presence of redox additives such as NaBH_4_ or AuHCl_4_ did not show any relevant effect on the composition of the obtained nanostructures, made of Zn.^[^ ^34^ ^]^ In that case, the Zn NPs were oxidized in water several ms up to hours after LAL, due to the different chemical reactivity compared to iron in Au–Fe nanoalloys. Indeed, all the Au–Fe samples of this study have been transferred in water after LAL to homogeneize the long‐term aging effects, while focusing only on the differences due to the laser ablation mechanism in the various liquid environments.

The time‐resolved experiments indicated almost no distinctive alteration in the dynamics of the cavitation bubble and of the shockwave when the additives were present. The only exception was represented by a decrease of 17–19% of the pressure at the front of the shockwave in the first ns after the absorption of the laser pulse in the presence of the O_2_‐evolving additives in water (H_2_O/O_2_) and ethanol (EtOH/O_2_), compared to pure liquids (H_2_O and EtOH). Since the other gas‐evolving additives (NaN_3_ and NaBH_4_) have also the effect of reducing the density of oxidising species (d_O_) by releasing gases as H_2_ or N_2_ (see Section S2, Supporting Information), the correlation between p_s_ and the fraction of substitutional iron in these samples is not meaningful as in the H_2_O/O_2_ and EtOH/O_2_ samples.

Figure 6b shows that p_s_ at 26 ns linearly correlates with the content of substitutional iron measured from the diffractograms of H_2_O, H_2_O/O_2_, EtOH and EtOH/O_2_ samples using Vegard law. A lower p_s_ in the first ns after laser ablation corresponds to a lower density at the interface between the plasma plume and the surrounding liquid, specifically in the thin supercritical liquid layer above the ablation spot.^[^ ^5^, ^6^, ^7^, ^8^, ^31^ ^]^ This implies a lower efficiency in the mixing of the metal species ejected from the target with the molecular compounds in the solution, which include the oxidising species.^[^ ^5^, ^6^, ^7^, ^8^, ^31^ ^]^ The time‐dependent pressure profile of a shock wave in an open environment, commonly used in shock wave propagation in air and explosion physics, is described by the adapted Friedländer equation:^[^ ^58^ ^]^

(1) $$p_{s}\;\left(t\right)=p_{s}^{max}\;\left(1-\frac{t}{t_{s}}\right)\exp\left(-\alpha \frac{t}{t_{s}}\right)$$

with *p_s_^max^* the value of the overpressure at time *t = 0* and α an empirical delay constant. The *p_s_* is 0 at *t_s_* and, for α = 1, reaches a negative minimum (*p_s_^min^*) at *2 t_s_* which is ‐13.5% of *p_s_^max^* (see Figure S17a, Supporting Information). Hence, a stronger initial blast wave results in a deeper negative pressure phase following the positive overpressure decay. For shock wave propagation in liquids, there is no corresponding analytical model as Equation (1). However, numerical simulations and direct measurements show similar behavior, with the same order of magnitude between the maximum overpressure and the negative pressure reached.^[^ ^54^, ^59^ ^]^ For instance, the early stage of the shock‐driven explosion of liquid‐water microdroplets was studied with the finite‐volume method, incorporating a high‐resolution discretization of the axisymmetric Euler equations, resulting in computed pressure wave profiles that are fully consistent with the time‐dependent overpressure described by the Friedländer equation.^[^ ^54^ ^]^ The measured *p_s_^min^* was ‐11% of the *p_s_^max^*, this negative pressure triggered the formation of a cavitation bubble. The negative pressure (tension) is expected to nucleate cavitation bubbles if it exceeds the tensile strength of the liquid.^[^ ^54^, ^60^ ^]^ In another study,^[^ ^59^ ^]^ the time‐dependent pressure of converging shock waves in water was studied with an optical‐fibre hydrophone, again resulting in a time‐dependent overpressure profile fully consistent with that described by the Friedländer equation. In this case, the measured *p_s_^min^* was ‐19% of the *p_s_^max^*. Under this evidence, a comparison of the pressure profiles in the four liquid environments (H_2_O, H_2_O/O_2_, EtOH, EtOH/O_2_) is reported in Figure S17b (Supporting Information) by exploiting the Friedländer equation. Since the *p_s_^min^* is more than 1 order of magnitude lower than *p_s_^max^*, the largest difference is observed at *p_s_^max^*, whereas the *p_s_^min^* are comparable. Consequently, in the four liquid environments, the strongest differences in the mixing of ablated matter with the liquid environment are expected when the pressure is maximum. In all cases, the negative pressure induced by the shock wave in our experimental conditions is an order of magnitude greater than what is required to nucleate the vapor phase and initiate cavitation.^[^ ^60^ ^]^ In fact, both experiments and MD calculations demonstrated that NPs nuclei, reactive target species, and reactive solution species (e.g., hydrogen and reactive oxygen species, ROS) are present in the plasma plume since its first appearance.^[^ ^5^, ^11^, ^33^, ^38^, ^39^ ^]^ Therefore, any variation of chemical composition at the plasma/solution interface already in the first ns of LAL is crucial for the final nanoalloy composition. It should be noted that the time‐resolved experiments were performed in ideal conditions for optimal observation of the experimental endpoints, in which liquid refreshing was applied to limit the interaction of laser pulses with NPs and persistent microbubbles. On the contrary, in static LAL, a higher concentration of persistent bubbles was observed in the presence of gas‐evolving solutes (Figure 6c). The presence of tiny gas bubbles in the liquid surrounding the ablation spot reduced the probability of interaction of the target and the ablated matter with the liquid solution. This is another mechanism that decreases the mixing efficiency between the metal species ejected from the target and the molecular compounds in the solution, which are responsible for iron oxidation and dealloying. The use of laser pulses with a duration > 5 ns, which overlap in time with the plasma plume and the first portion of the ablated matter,^[^ ^4^, ^5^, ^31^ ^]^ is expected to further favor the mixing of reactive target and solution species, thus being more sensitive to any physical phenomena that reduce their mixing. The critical role of mixing between target and solution species in the early stage of LAL was previously hypothesized by looking at the composition of Au–Fe nanoalloys obtained by laser ablation of a Fe/Au/glass thin film in water and in ethanol.^[^ ^27^ ^]^

### 3.3 Additional Factors Influencing LAL of Au–Fe NPs

According to the results summarized in Table S4 (Supporting Information), different laser pulse durations are associated with different atomic structures of the Au–Fe NPs. The ns laser pulses have been intensely exploited for the synthesis of Au–Fe nanoalloys and other types of nanoalloys. The fs and ps lasers are also frequently used for the production of Au–Fe and other nanoalloys, even at the largest productivity reported so far.^[^ ^1^, ^3^, ^61^ ^]^ However, it should be noted that ns lasers currently offer the best NPs investment‐specific productivity.^[^ ^61^ ^]^ MD simulations suggested that the region of interconnection between the ablated matter and the surrounding liquid species has a lower extension in space and time when the pulse duration is lower.^[^ ^6^, ^7^ ^]^ This generally results in lower chemical reactivity of the produced NPs with the liquid environment and a final stoichiometry more similar to the original target with fs and ps pulses than with ns ones.^[^ ^23^, ^62^, ^63^ ^]^ Besides, fs and ps pulses avoid overlap of the laser beam with the plasma plume, a phenomenon which can promote further the reactivity of the ablated matter with the liquid environment and is observed when the laser pulse duration exceeds hundreds of ps.^[^ ^6^, ^7^, ^62^, ^63^, ^64^ ^]^ Although this is an advantage for the production of nanoalloys from compositionally homogeneous targets, it has also been demonstrated that ultrashort pulses are less effective for the achievement of nanoalloys from heterogeneous targets.^[^ ^23^, ^62^, ^63^ ^]^ In fact, the ns pulses allow better elemental mixing in the case of targets made of sintered powders or composed of immiscible elements, which may be heterogeneous at the microscopic scale.^[^ ^23^, ^62^, ^63^ ^]^

Pulse fluence is less frequently used as a parameter in the LAL synthesis because it is usually constrained in a relatively tight range between the ablation and the liquid breakdown thresholds. According to experimental investigations, particle size increases with laser fluence.^[^ ^65^ ^]^ MD simulations indicated that this is due to the increase in the thickness of the top transient spongy structure of interconnected liquid regions in the target, which subsequently decomposes leading to the formation of large NPs.^[^ ^6^, ^7^ ^]^ Hence, when the fluence remains below the threshold for liquid breakdown, the increase of pulse fluence is expected to favour the formation of large multimetallic NPs, whose inner composition is typically less influenced by the external chemical environment.^[^ ^6^, ^7^, ^62^ ^]^ Besides, the shock front pressure increases with the pulse power density.^[^ ^66^ ^]^

The colloidal stability and the reirradiation of NPs during LAL have also been considered as factors potentially affecting the atomic structure of the Au–Fe NPs. The colloidal stability of the eight samples was investigated by dynamic light scattering (DLS) and surface z‐potential. The analysis was performed on the as‐synthesized colloids and the final samples resuspended in water (Figure S18a, Supporting Information). In the original synthetic environments, the hydrodynamic diameters and the z‐potentials of the NPs show no difference among the four samples which can be related to their different structure or composition (Figure S18b, Supporting Information). In water, the hydrodynamic size is lowest in the H_2_O/N_2_ sample (15 ± 3 nm) and largest in the H_2_O/H_2_ one (92 ± 23 nm), with H_2_O (47 ± 9 nm) and H_2_O/O_2_ (48 ± 8 nm) in between, whereas the z‐potentials do not follow this trend, with the maximum value for the H_2_O sample (−32 ± 14 mV) and lowest in the H_2_O/N_2_ one (‐9 ± 7 mV). In ethanol, the gas‐evolving additives are associated with hydrodynamic sizes of hundreds of nm, compared to 23 ±3 nm for the EtOH sample (Figure S18a, Supporting Information). However, the z‐potentials are larger in EtOH/O_2_ and EtOH/H_2_ (Figure S18b, Supporting Information), indicating the absence of a specific correlation with the hydrodynamic diameter. After resuspension of the NPs in pure water, the samples with the strongest evidence of iron oxidation (H_2_O and H_2_O/N_2_, followed by H_2_O/O_2_) also have the largest hydrodynamic size (Figure S18c, Supporting Information). However, the z‐potential does not follow this trend (Figure S18d, Supporting Information) and is homogeneous among water (H_2_O: −28 ± 4 mV; H_2_O/O_2_: −27 ± 5 mV; H_2_O/H_2_: −20 ± 4 mV; H_2_O/N_2_: −27 ± 5 mV) and ethanol (EtOH: −11 ± 3 mV; EtOH/O_2_: −17 ± 3 mV; EtOH/H_2_: −15 ± 3 mV; EtOH/N_2_: −20 ± 3 mV) samples.

Overall, the colloidal stability of the NPs does not appear as related to the alloy composition. Therefore, the structural changes observed in the eight samples are attributed to physical‐chemical processes occurring in an early stage of the NPs formation, where steady‐state colloidal stability has no effects. Since the EtOH/H_2_ NPs have a dendritic structure according to TEM analysis, this is attributed to the limited surface oxidation and presence of an Au skin. In similar cases, the addition of polymeric stabilizers during the LAL was a viable strategy to prevent NPs aggregation.^[^ ^12^, ^13^, ^14^, ^26^ ^]^

The effect of NPs reirradiation was tested by a dedicated experiment (see Figure S19a, Supporting Information) applying the same parameters, duration, and conditions of the LAL synthesis but using as the liquid solution the Au–Fe colloid previously synthesized in water (H_2_O) or ethanol (EtOH). Then, H_2_O_2_ was added to the solution just before the reirradiation, and the bulk target was removed in order to avoid the mixture of freshly generated NPs with the pristine one subjected to the laser irradiation. This experiment stresses the reirradiation conditions achievable during a typical batch synthesis LAL, where the overlap of laser‐generated NPs and the incident laser beam has a much shorter duration, in particular, due to the convective flux around the laser spot. The products were collected by centrifugation and resuspended in water as in the usual procedure and, finally, analyzed with XRD. The results (Figure S19b,c, Supporting Information) show that reirradiation did not induce alloying in the water samples, while it only contributed to dealloying in the ethanol samples. Therefore, reirradiation is not responsible for the better alloying observed in the H_2_O/O_2_ and EtOH/O_2_ samples compared to the H_2_O and EtOH ones.

### 3.4 General Overview

Overall, by joining the structural analysis of the Au–Fe nanoalloys with the time‐resolved investigation of the LAL dynamics, a double effect can be associated with the presence of gas‐evolving solutes: i) the chemical contribution due to the redox species released in the synthesis environment (either O_2_, H_2_ or the inert N_2_); ii) a dominating physical effect which reduced the mixing of ablated matter with solution species and ROS responsible for dealloying. The combination of these two effects led to the variety of ultrastructures observed in laser‐generated Au–Fe nanoalloys. Some of the nanostructures observed in our experiment as a function of the density of oxidative species (d_O_) and shock front pressure (p_s_) are represented in Figure 6a.

Significant efforts have been deployed to date on the ex situ investigation of LAL products and the in situ investigation of cavitation bubbles, as well as the in silico study of LAL physics. Similar efforts are desirable in the future regarding the shock front pressure and along the hypersurface of LAL parameters in the presence of gas‐evolving solutes, which includes thousands of possible combinations among laser pulse wavelength, fluence, duration, repetition rate, static or fluxed cell synthesis, type of solvent, concentration and type of gas evolving solutes, the target composition and the presence of stabilizers for the NPs. For instance, the improvement of MD modelling to better capture the first tens of ns after laser pulse absorption, with particular emphasis on the mixing between target and solution species and on the chemical processes taking place thereof. Concurrently, in the future there is a need for the advancement of in situ synchrotron techniques with ultrafast time resolution to further identify the chemical trajectories in the LAL of metastable nanoalloys and nanomaterials. In this way, one can expect further refinement in the control of the products composition and ultrastructure and the possible transition to large scale production of nanomaterials with complex morphology.

## 4 Conclusion

The LAL synthesis of nonequilibrium Au–Fe nanoalloys was studied in the presence of gas‐evolving solutes, aiming at improving the understanding of the phenomena involved and the control over the local atomic structure of metastable nanophases. The process led to various alloy compositions and textures at the nanometer scale, such as Janus, core‐crescent, homogeneous and heterogeneous alloys, which became fully evident only with high‐resolution analytical TEM techniques targeting the ultrastructure at the single NPs level. The entire process simply involved the LAL of a unique bulk metal plate immersed in green liquids containing common gas‐evolving solutes. To clarify the relation between the products and the synthetic environment, a dedicated time‐resolved investigation of the entire and early stage of the LAL was performed. Results indicated that gas‐evolving solutes did not alter the dynamics of the cavitation bubble, compared to pure liquids. Concerning the shockwave, the only difference found was a reduction of 17–19% of the pressure at the shockwave front just after the absorption of the laser pulse when H_2_O_2_ was present. This reduction linearly correlates with the content of substitutional iron in the nanoalloys, pointing to the pivotal role of reducing the pressure at the shockwave front to preserve the composition of metastable multielement compounds. Besides, a higher concentration of persistent microbubbles was observed when gas‐evolving solutes were used. Both phenomena reduce the mixing of ablated matter with the ROS generated in the supercritical liquid layer, which are active components in the thermodynamic and chemical pathways to dealloying. Such mechanisms explain the formation of Au–Fe nanoalloys in H_2_O/O_2_ and EtOH/O_2_ with higher efficacy than in the corresponding pure solvents. This outcome demonstrates that LAL was exploited to mold the ultrastructure of metastable nanoalloys composed of elements in great demand for plasmonic, magnetic, catalytic, and biomedical applications, which is among the most advanced goals of modern nanotechnology.

## Conflict of Interest

The authors declare no conflict of interest.

## Supporting information



Supporting Information

## Data Availability

The data that support the findings of this study are available from the corresponding authors upon reasonable request.
