# Supplementary material for: Time‐Resolved Dynamics of Laser Ablation in Liquid with Gas‐Evolving Additives: Toward Molding the Atomic Structure of Nonequilibrium Nanoalloys
Source: Adv Sci (Weinh). 2025 Apr 26;12(21):2416035. doi: 10.1002/advs.202416035 (PMC12140356; doi:10.1002/advs.202416035)
Supplement: Supplementary file 1 — Supporting Information [file ADVS-12-2416035-s001.pdf]

ADVANCED SCIENCE
Open Access

Supporting Information

for *Adv. Sci.*, DOI 10.1002/advs.202416035

Time-Resolved Dynamics of Laser Ablation in Liquid with Gas-Evolving Additives: Toward Molding the Atomic Structure of Nonequilibrium Nanoalloys

Vito Coviello, Catherine Reffatto, Mehdi W. Fawaz, Benoit Mahler, Arnaud Sollier, Bratislav Lukic, Alexander Rack, David Amans and Vincenzo Amendola**

Supporting Information

Time-Resolved Dynamics of Laser Ablation in Liquid with Gas-Evolving Additives: Towards Molding the Atomic Structure of Nonequilibrium Nanoalloys

Vito Coviello, Catherine Reffatto, Mehdi W. Fawaz, Benoit Mahler, Arnaud Sollier, Bratislav Lukic, Alexander Rack, David Amans, and Vincenzo Amendola**

Vito Coviello, Catherine Reffatto, Vincenzo Amendola*
Department of Chemical Sciences, Università di Padova, via Marzolo 1, I-35131 Padova, Italy
E-mail: vincenzo.amendola@unipd.it

Mehdi W. Fawaz, Benoit Mahler, David Amans,*
Université Claude Bernard Lyon 1, UMR 5306 CNRS, Institut Lumière Matière, Villeurbanne, F-69100, France
E-mail: david.amans@univ-lyon1.fr;

Arnaud Sollier
CEA, DAM, DIF, F-91297 Arpajon, France
Université Paris-Saclay, CEA, Laboratoire Matière en Conditions Extrêmes, F-91680 Bruyères- Le-Chatel, France

Bratislav Lukic, Alexander Rack
ESRF-The European Synchrotron, CS40220, F-38043 Grenoble, France

Contents		***page***
**1.**	**Materials and methods**	**S2**
**2.**	**Decomposition of gas-evolving solutes**	**S7**
**3.**	**Tables and Figures**	**S8**
**Table S1**	**Synthetic conditions**	**S8**
**Table S2**	**Rietveld refinement**	**S8**
**Table S3**	**Overview of applications for Au-Fe nanostructures**	**S9**
**Table S4**	**Overview of Au-Fe nanostructures obtained with different LPSC techniques**	**S10**
**Figure S1**	**Yield of NPs synthesis after resuspension in pure water**	**S11**
**Figure S2**	**XRD analysis**	**S11**
**Figure S3**	**TEM images and histograms**	**S12**
**Figure S4**	**RA calculation**	**S13**
**Figure S5**	**HRTEM and EDX analysis of sample H_2O and H_2O/N_2**	**S14**
**Figure S6**	**HRTEM and FFT analysis of sample H_2O**	**S15**
**Figure S7**	**HRTEM and FFT analysis of sample H_2O/N_2**	**S16**
**Figure S8**	**EDX, HRTEM and FFT analysis of samples H_2O/O_2 and H_2O/H_2**	**S17**
**Figure S9**	**EDX of samples EtOH, $EtOH/N_2$, $EtOH/O_2$ and $EtOH/H_2$**	**S18**
**Figure S10**	**EDX, HRTEM and FFT analysis of samples EtOH**	**S19**
**Figure S11**	**EDX, HRTEM and FFT analysis of samples $EtOH/N_2$**	**S20**
**Figure S12**	**EDX, HRTEM and FFT analysis of samples $EtOH/O_2$**	**S21**
**Figure S13**	**EDX, HRTEM and FFT analysis of samples $EtOH/H_2$**	**S22**
**Figure S14**	**Plot of R_{MAX} versus composition**	**S23**
**Figure S15**	**Cavitation bubble pressure at R_{MAX}**	**S23**
**4.**	**Thermodynamic model for Gibbs free energy in Au-Fe NPs**	**S24**
**Figure S16**	**Plot of Gibbs free energy for Au-Fe NPs with SS or CS morphology**	**S25**
**Figure S17**	**Plot of shock wave pressure with the Friedländer equation**	**S26**
**Figure S18**	**DLS and z-potential of Au-Fe samples**	**S27**
**Figure S19**	**Effect of reirradiation on Au-Fe samples**	**S28**
**5.**	**References**	**S29**

1. Materials and methods.

LAL synthesis of Au-Fe NPs.

LAL synthesis of Au-Fe NPs was performed by focusing 1064 nm (6 ns, 50 Hz) pulses of a Nd:YAG laser with a lens (f 10 cm) at a fluence of 10 J/cm^2 on the Au:Fe (72:28 atomic ratio) alloy target (>99.9 % pure, from Mateck) placed at the bottom of a glassy batch chamber mounted on a motorized XY scanning stage (Standa). Eight different synthetic environments, as summarized in Table S1, have been tested by combining distilled water (H_2O) or ethanol (EtOH, HPLC grade, >99.9%, Sigma-Aldrich) with H_2O_2 (0.01% Vol/Vol of H_2O_2 stabilized 30%, from Sigma Aldrich), $NaBH_4$ (0.1 mg/mL, from Sigma Aldrich, purity >98%), and NaN_3 (0.1 mg/mL, from Supelco® aqueous solution 0.05%). The ablation cell was kept under Ar atmosphere by bubbling the inert gas into the liquid solution since 5' before the synthesis.

Post-LAL, all the colloidal samples were washed three times with distilled water by centrifugation at 2000 rcf for 30' and finally resuspended in distilled water. This procedure was adopted to remove any difference in the structure or composition of the NPs due to their dispersion in the liquid or the solute *after* the LAL process. The yield of the process was 98.97 ± 0.06 % for H_2O, 99.91 ± 0.04 % for H_2O/O_2, 95.9 ± 0.4 % for H_2O/H_2, 99.90 ± 0.01 % for H_2O/N_2, 83.1 ± 0.7 % for EtOH, 99.4 ± 0.7 % for EtOH/O_2, 99.0 ± 0.5 % for EtOH/H_2 and 99.3 ± 0.4 % for EtOH/N_2, as assessed by UV-vis spectroscopy on the colloids as-synthesized and after centrifugation (Figure S1).

Characterization.

UV-visible absorption spectroscopy was performed with a JASCO V770 UV−vis−NIR spectrometer in 2 mm quartz cells for each sample just after the synthetic procedure described in Table S1. Powder XRD analysis was performed with a Panalytical XPert 3 Powder diffractometer equipped with a Cu tube (40 kV, 40 mA), a BBHD mirror, a spinner, and a PlXcel detector. The samples were deposed on Si zero-background substrates by drop-casting and drying at room temperature. The diffractograms were analyzed with TOPAS Academic V6 (Bruker AXS) using the COD databases. Rietveld refinement was performed after fitting the background with a Chebyshev function. The shape of the reflections was modelled through the fundamental parameter approach incorporated in the program, separating the instrumental and the sample contributions. Fit indicators R_{wp}, R_{exp}, and GoF (Goodness of Fit) were used to assess the quality of the refined structural models.

To account for the two different populations of particles in most samples, the average composition of the nanoalloys has been calculated by the mass-weight of the two populations. For each population, the gold atomic content has been estimated using Vegard law based on

literature data in the composition range from pure Au to Au:Fe 60:40,[1] resulting in Equation S1, and then averaged for its relative contribution to the refinement.

$$Au\ at.\% = \frac{a-4.07754}{-0.32916} \qquad \text{(Equation S1)}$$

Samples for TEM analysis were prepared by evaporating the colloids on a carbon film supported on copper grids, 400 mesh size. Bright-field TEM analysis was performed with a FEI Tecnai G2 12 operating at 100 kV and equipped with a TVIPS CCD camera. The TEM size distributions have been calculated by accounting for at least 500 NPs per sample using the ImageJ particlesizer plugin implemented in the Fiji software and calculating the size as the diameter of the circle with an equivalent area, after setting a convexity threshold value at 0.99 and a size threshold value at 1.5 nm.[2] The NPs were counted from at least 5 different images per sample. TEM image segmentation was performed with an ImageJ plug-in (trainable weka segmentation),[3] Two representative images for each sample have been selected for the segmentation analysis.

HRTEM analysis was carried out with a JEOL 2100HT microscope operating at 200 KV using a Gatan Orius 200 camera. Energy Dispersive X-ray (EDX) spectroscopy was performed using an SDD Xmax80 detector from Oxford Instruments. HR-STEM-EDX analyses have been performed on a JEOL JEM-ARM200F Cold FEG operating at 200 kV equipped with an SDD CENTURIO-X EDX analyzer. The EDX elemental mapping was performed with a probe spatial resolution of 1 nm and one spectrum acquired each nm^2.

The morphology and microstructure of the samples were further characterized by transmission electron microscopy (TEM) and high-angle annular dark-field (HAADF) scanning transmission electron microscopy (HAADF-STEM) using a Cold-FEG S/TEM JEOL JEM F200 operating at 200 kV. Elemental analysis and mapping were performed using a JEOL 100 mm^2 silicon drift energy EDX spectrometer.

Dynamic light scattering (DLS) and z-potential analysis were performed with a Malvern Zetasizer Nano ZS using ZEN0112 (DLS) or DTS1070 (z-potential) cells. The refractive index of the solvent (water or ethanol) was set for the analysis. For the dispersed material, the refractive index of gold was used.

Shadowgraph imaging.

Shadowgraph imaging experiments have been conducted using a passively Q-switched Nd-YAG laser (1064 nm, 1 kHz), according to the setup equivalent to the sketch in Figure 4b. The beam is amplified using a laser gain module (Taranis module), which consists of a diode-pumped Nd:YAG single-crystal fibre,[4] resulting in a laser pulse (0.5 ns, 1.5 mJ/pulse, 1 kHz) focused (f 160 mm) at a fluence of 12 J/cm^2 on the Au-Fe target placed 1 cm under the solvent

level to keep the laser conditions stable between each pulse. A liquid flux was applied close to the ablation spot to keep the ablation area clean from persistent microbubbles and prevent the ablated material from absorbing part of the laser light, attenuating the energy delivered to the target. The shadowgraph images for monitoring the dynamics of the cavitation bubbles (μs time resolution) have been collected with an ultrafast camera Phantom v711 (from Vision Research) coupled with a Navitar 2.0X zoom lens system. A pattern of ultrabright LEDs is used for illumination. The camera rate is 210000 frames/s and recorded images with a resolution of 128 x 128 pixels. A spatial resolution of 42 μm is achieved in our experimental conditions. A minimum of 35 bubbles have been analyzed for each synthetic condition with the time 0 set by the image showing the native plasma plume since the plasma lifetime is shorter than the time between the two frames.[5]

Cavitation bubble dynamics.

Among the various models available, the bubble dynamics was described analytically using the commonly adopted Rayleigh-Plesset (RP) theory.[5]

The RP equation is defined as:

$$R\ddot{R} + \frac{3}{2}\dot{R} = \frac{1}{\rho}\left[P_B(t) - P_l - \frac{2\sigma}{R} - \frac{4\eta\dot{R}}{R}\right] \qquad \text{(Equation S2)}$$

Where R, P_B, P_l, σ, η and ρ are respectively the radius of the bubble, the bubble pressure, the pressure of the surrounding liquid, the surface tension, and the dynamic viscosity and the density of the liquid. As reported by Lam et. al,[5] assuming adiabatic evolution, the equation can be reduced to equation S3, which can be used to compute the time evolution of the bubble radius:

$$\dot{R}^2 = \frac{2}{3\rho}\left(\frac{1}{1-\gamma}P_B - P_l\right) + \frac{C_1}{R^3} \qquad \text{(Equation S3)}$$

Here γ is the heat capacity ratio.

In the RP model, the pressure of the vapour inside the bubble is evaluated according to a previously described procedure,[5] by rearranging Equation S3 as:

$$P_B = (1-\gamma)\left\{P_l + \frac{3\rho}{2}\left(\dot{R}^2 - \frac{C_1}{R^3}\right)\right\} \qquad \text{(Equation S4)}$$

where C_1 is an integration constant that can be determined at the maximum size where the 1st derivative of the bubble radius is null. In that case, it is possible to solve

Equation S4 as

$$C_1 = \frac{2R_{max}^3}{3(\gamma-1)}\left(\frac{\gamma P_l}{\rho} + R_{max}\ddot{R}_{max}\right) \qquad \text{(Equation S5)}$$

The dynamics of the bubbles have been fitted with Equation S3 using the finite difference method (Euler method), as well as the pressure inside the bubbles has been estimated with Equation S4-S5, using a Python 3.0 script.

The Euler method starts from the maximum radius R_{max} and proceeds independently step by step towards the initial and the collapse time. The reason for starting from R_{max} arises from the stability that the bubbles have at that point, which minimizes the uncertainty given by the Euler method. In the calculation procedure, the heat capacity ratio and the liquid pressure are free parameters. Indeed, the RP model does not account for liquid compressibility, and generally, its effect is accounted for by considering the liquid pressure as a free parameter.

The analysis was performed in the hypothesis of adiabatic conditions leading to symmetric bubble growth and collapse over time. However, this behaviour is not observed in all measurements and the non-symmetric behaviour was accounted for by separately fitting the growth and the collapse stages.

The Rayleigh collapse time (t_R) used for the comparison of bubble dynamics among different samples, by resorting to the reduced R/R_{MAX} and t/t_{MAX} quantities, is defined as:

$$t_R = 0.91468 R_{max}\sqrt{\frac{\rho}{p_\infty}} \qquad \text{(Equation S6)}$$

Where p_∞ is the external pressure at an infinite distance from the bubble, in this case, considered as the atmospheric pressure.

Shockwave and early cavitation bubble dynamics.

The shadowgraph images for monitoring the dynamics of the shock wave (ns time resolution) were collected with a setup built at the ESRF synchrotron facility (Grenoble, France). Images were recorded with a multi-channel framing SIMX Camera equipped with 16 iCCD sensors able to record simultaneously 16 HR images with a different delay time. The light source used for the imaging is a pulsed diode laser light source (Cavilux from Cavitar). The LAL was performed with a Nd:YAG laser (532 nm, 10 Hz, 100 mJ/pulse, 5 ns) focused (f 50 mm) at a fluence of 44 J/cm^2. Laser parameters were set for optimal observation of the shockwave and the cavitation bubble and compatibly with what was available at the ESRF facility. The images are taken with a delay time of 13 ns, of which 5 ns is the gating time of the camera and 8 ns is the delay time set between each acquisition. The liquid solution was changed for each acquisition to avoid the extinction of the laser beam by NPs and persistent microbubbles, with a consequent reduction of the energy conveyed to the target. Five measurements have been carried out for each type of liquid environment.

Shockwave dynamics model.

The pressure at the shockwave front was linked to its velocity, directly measured from the shadowgraph images, using the Hugoniot curve for liquids, which describes the relationship between the initial and final states of a fluid undergoing a shockwave.[6] Thanks to the conservation of the momentum at the shock front,[7] the pressure at the shock front is linked to the particle velocity (u_p) and the shock wave velocity (u_s) by:

$$p_s = p_\infty + \rho\, u_s u_p \quad \text{(Equation S7)}$$

The problem is, thus, reduced to the calculation of u_p from u_s. The Universal Liquid Hugoniot (ULH) equation developed by Woolfolk and Shaw has been demonstrated to well describe the shockwaves for different liquids according to:[8]

$$u_s = 1.37c_0 - 0.37c_0 \exp\left(-\frac{2u_p}{c_0}\right) + 1.62u_p \qquad \text{(Equation S8)}$$

where c_0 refers to the ambient condition sound speed. According to this equation, pressures of the shock front have been calculated at time 0 and 26 ns (corresponding to the first experimental point where the shockwave can be identified).

As a further verification of the pressure values, a comparison was done between the results of the ULH equation and the ones derived from the Rice and Walsh equation of states (EOS) for water,[9] resulting in a good agreement.

Statistical analysis

TEM size histograms represent the averaged Feret size measured from a minimum of 500 nanoparticles per sample arising from at least 5 TEM images per sample. The mean value has been used, and the error is defined as standard deviation (SD). The TEM segmentation was performed on at least two images per sample to include at least 300 NPs. To transform the obtained green, red, and violet channels of the images, the split of the channel was done in order to isolate the single one, then the image was converted to black and white, and the area was calculated with the ImageJ software, the reported value is the average, and the error is considered as the maximum semi-dispersion. Cavitation bubbles' mean size and lifetime were obtained by analysing a minimum of 35 bubbles per sample. The error bars correspond to the two-sided level of confidence at 95% on average over 35 independent measurements. The experimental shock front positions (d) were obtained from the average among 5 independent measurements per sample, considering the SD as the error. A polynomial fit of the 4th order was used to fit the position, and the goodness of the fits was estimated with the coefficient of determination R^2, resulting larger than 0.997 for all the cases. The u_s is then obtained from the first derivative of the polynomial fit. The reported velocity errors are calculated considering the interval of confidence on the position. The p_s (pressure of the shock front) was estimated from

u_s according to Equation S7, and the errors on p_s were obtained from the quadratic error propagation formula.

2. Decomposition of gas-evolving solutes.

The following decomposition reactions occur for H_2O_2 and $NaBH_4$ in presence of water:[10–14]

$2\ H_2O_2 \longrightarrow 2\ H_2O + O_2$

$Na^+\ BH_4^- + 2\ H_2O \longrightarrow Na^+ + BO_2^- + 4\ H_2$

Since the ethanol used in this study was not anhydrous, the same decomposition reactions apply also in this solvent.

Regarding NaN_3, the N_3^- ion is relatively stable in water[15–18] and, at the high temperature reached during LAL, it can decompose as

$2\ N_3^- + 2\ H_2O \longrightarrow 3\ N_2 + H_2 + 2\ OH^-$

In non-anhydrous ethanol, the N_3^- ion is less stable and the above decomposition reaction takes place at lower temperatures.[15–18] Alternatively, ethanol molecules can be reduced to enolates by N_3^- decomposition.[15–18]

Temperature is identified as the main factor triggering the decomposition of the three additives.[10–18] Hence, the high temperature reached in proximity to the ablation spot promotes the decomposition of solutes and gas evolution. Additionally, according to literature,[10–18] the presence of metal NPs can further catalyze the decomposition reactions.

3. Tables and Figures.

Sample Name	Target	Solvent	Additive	Other conditions	Sample collection
H_2O	Au-Fe 72-28 at.%	H_2O (12 mL)	-	Argon flux 45 min LAL Repeated 2 times	3 x 2000 rcf 30 min each with H_2O
H_2O/O_2			H_2O_2 (0.1 μL/mL at 30% v/v)		
H_2O/H_2			$NaBH_4$ (0.1 mg/mL)		
H_2O/N_2			NaN_3 (0.1 mg/mL)		
EtOH		EtOH (12 mL)	-		
$EtOH/O_2$			H_2O_2 (0.1 μL/mL at 30% v/v)		
$EtOH/H_2$			$NaBH_4$ (0.1 mg/mL)		
$EtOH/N_2$			NaN_3 (0.1 mg/mL)		

Table S1. Synthetic conditions. Summary of the synthetic conditions for the eight samples.

	Phase	Amount (wt. %)	Crystallite Size (nm)	Lattice parameter (Å)	Average substitutional Fe from Vegard law (at. %)
H_2O	FCC I	8 ± 2	65.1	4.032 ± 0.002	**3.78 ± 0.3**
	FCC II	92 ± 2	10.1	4.068 ± 0.002	
H_2O/O_2	FCC I	58 ± 4	26.1	4.0180 ± 0.0002	**14.5 ± 0.1**
	FCC II	42 ± 4	3.9	4.0464 ± 0.0001	
H_2O/H_2	FCC I	35 ± 2	54.6	4.0265 ± 0.0006	**9.04 ± 0.06**
	FCC II	64 ± 2	5.8	4.0596 ± 0.0007	
H_2O/N_2	FCC I	100	12.3	4.069 ± 0.001	**2.44 ± 0.01**
EtOH	FCC I	100	16.2	4.0372 ± 0.0002	**12.26 ± 0.01**
$EtOH/O_2$	FCC I	60 ± 10	35.0	4.0030 ± 0.0001	**19.7 ± 03**
	FCC II	40 ± 10	5.2	4.0298 ± 0.0006	
$EtOH/H_2$	FCC I	52 ± 9	36.7	3.9980 ± 0.0001	**16.3 ± 0.3**
	FCC II	48 ± 9	5.2	4.0496 ± 0.0005	
$EtOH/N_2$	FCC I	49 ± 2	8.0	4.0244 ± 0.0006	**20.01 ± 0.06**
	FCC II	51 ± 2	8.7	3.9994 ± 0.0003	

Table S2. Rietveld refinement. Results of Rietveld refinement for the eight samples, crystallite size obtained from Scherrer equation, and Fe at% obtained by applying the Vegard law to the mass-weighted lattice parameter.

Structure	Found in samples	Application described in literature
Au-FeOx<br>Janus/heterostructures	H_2O<br>H_2O/N_2	MRI + CT + PA + SERS[19]<br>MRI + SERS + PA[20]<br>MRI + optical imaging [21] (Fe3O4@Au)<br>MRI + CT + PTT + PA[22]<br>MRI+ PTT+ PA [23]<br>Drug-delivery + PTT [24]<br>PA + PTT[25]<br>Magnetic hyperthermia [26]<br>Magnetic hyperthermia + PTT [27] [28]<br>Ferroptosis + MRI[29]<br>Ferroptosis [30]<br>Drug-delivery+radiotherapy [31]<br>Drug delivery [32–34]<br>Biocompatible scavengers [35]<br>Magnetic separation [36]<br>Cell labelling [37]<br>Biosensing [38]<br>Electrochemical sensing [39]<br>Optical Sensing [40–43]<br>Surface-enhanced fluorescence [44]<br>SERS [45–47]<br>CO oxidation [48,49]<br>Methanol oxidation [50]
Au-Fe/FeOx-shell	H_2O/O_2<br>H_2O/H_2<br>EtOH<br>$EtOH/N_2$<br>$EtOH/O_2$	Drug-delivery[51]<br>Photothermal therapy [51]<br>Magneto-optics[52,53]<br>Oxygen evolution reaction [54]<br>SERS [55,56]
Au-Fe with Fe-rich domains	H_2O/O_2<br>H_2O/H_2	Drug delivery + MRI + Hyperthermia [55]<br>MRI [57]<br>Theranostics [58]<br>Magneto-optics[59,60]
Au-Fe homogeneous	EtOH<br>$EtOH/N_2$<br>$EtOH/O_2$	MRI+CT+SERS labelling[61]<br>MRI+CT+biodegradability[62]<br>MRI[63,64]<br>Ferroptosis[65]<br>Magnetic hyperthermia + drug delivery [66,67]<br>Magnetic hyperthermia [68]<br>Chiro-magneto-optics [69]<br>SERS[70]<br>Optical sensing [71]<br>Oxygen evolution reaction[72]<br>CO oxidation [73]<br>Oxidation catalysis[74]
Au-Fe/Au-shell	$EtOH/H_2$	Magnetic-field enhanced SERS[75,76]<br>CO_2 reduction [77]

Table S3. Overview of applications for Au-Fe nanostructures. The applications of Au-Fe nanostructures are summarized according to the different morphology and atomic structure. Abbreviations are CT: X-ray computed tomography, MRI: Magnetic resonance imaging, SERS: Surface-enhanced Raman scattering, PTT: Photothermal Therapy, PA: Photoacoustic imaging.

Synthetic Parameters						NPs information		Ref.
**LSPC technique**	**Starting Material**	**Target Composition**	**Laser parameters**	**Solvent**	**Solute/Gas**	**NPs Structure**	**Nanoalloy Composition**	
LAL	Alloy Target	Au 72-Fe28	1064 nm<br>6 ns<br>65 mJ cm^{-2}<br>50 Hz	Ethanol	-	Au-Fe@FeOx	Au-Fe 88-12 at%	This Work
					H_2O_2 0.1 µL/mL 30% v/v	Au-Fe@FeOx	Au-Fe 80-20 at%	
					$NaBH_4$ 0.1 mg/mL	Au-Fe@Au	Au-Fe 84-16 at%	
					NaN_3 0.1 mg/mL	Au-Fe@FeOx	Au-Fe 80-20 at%	
				H2O	-	Fe-doped Au@FeOx (Shell, Crescent, Janus)	Au-Fe 96-4 at%	
					H_2O_2 0.1 µL/mL 30% v/v	Fe-rich clusters in Au-Fe@FeOx	Au-Fe 86-14 at%	
					$NaBH_4$ 0.1 mg/mL	Fe-rich clusters in Au-Fe@FeOx	Au-Fe 91-9 at%	
					NaN_3 0.1 mg/mL	Fe-doped Au@FeOx (Shell, Crescent, Janus)	Au-Fe 98-2 at%	
		Fe-Au 44-56 at%	800 nm, 120 fs, 0.3 mJ, 5KHz	Methyl Methacrylate	-	Fe@Au + Au-Fe	Au-Fe 53-47 at%	[78]
			1064 nm, 10 ps, 0.16 mJ	Acetone				
			1064 nm, 8 ns, 0.8 mJ					
			800 nm, 120 fs, 0.3 mJ, 5KHz	Deionized Water		$Au@Fe_3O_4$		
		Fe-Au 10-90 at%	1064 nm<br>10 ns<br>3.85 mJ cm^{-2}<br>15 kHz	Acetone	-	Au-Fe	Fe-Au 10-90 at%	[79]
		Fe-Au 20-80 at%				Au-Fe	Fe-Au 20-80 at%	
		Fe-Au 35-65 at%				Au-Fe@Au (5 %) + Au-Fe (95 %)	Fe-Au 35-65 at%	
		Fe-Au 50-50 at%				Au-Fe@Au (73 %) + Au-Fe (27 %)	Fe-Au 50-50 at%	
		Fe-Au 65-35 at%				Au-Fe@Au (78 %) + Au-Fe (22 %)	Fe-Au 65-35 at%	
		Fe-Au 80-20 at%				Au-Fe@Au (85 %) + Au-Fe (15 %)	Fe-Au 80-20 at%	
		Fe-Au 90-10 at%				Au-Fe@Au (92 %) + Au-Fe (8 %)	Fe-Au 90-10 at%	
		Fe-Au 27-73 at%	1064 nm, 6 ns, 65 mJ cm^{-2}, 50 Hz	Ethanol	-	Au-Fe	Au-Fe 83-17 at%	[80]
							Au-Fe 80-20 at%	
		Fe-Au 50-50 at%	1064 nm, 10 ps, 3.81 mJ cm^{-2}, 100 kHz	Acetone	-	Fe@Au-Fe	Au-Fe 85-15 at% (shell)	[81]
		Fe-Au 80-20 at%	1064 nm, 8 ns<br>3.85 mJ cm^{-2}, 15 kHz	Acetone or 3-pentanone		Au-Fe@Au-Fe@@Fe3O4	Au-Fe 10-90 at% (core) + Au-Fe 50-50 at% (shell)	
						Nested core-shell Au-Fe@Fe@Au-Fe	Au-Fe 76-34 at% (core) + Au-Fe 97-3 at% (shell)	
		Fe-Au 27-73 at%	1064 nm, 6 ns<br>65 mJ cm^{-2} , 50 Hz	Ethanol	-	Au-Fe	Au-Fe 90-10at%	[82]
					H2O2			
					Ar			
					CO2			
					N2			
				Ethanol /H2O	-	Au-Fe@FeOx	Au-Fe 87-13 at%	
				H2O	H2O2	Au-Fe@FeOx	Au-Fe 97-3 at%	
	Thin film Fe-Au Alloy	Fe-Au 50-50 at%	1064 nm, 6 ns, 22.6 mJ	Acetone	-	Au-Fe@Au (87 %) + Au-Fe (13 %)	Au-Fe 50-50 at%	[83]
	Thin film Fe/Au/Glass					Au-Fe@Au (78 %) + Au-Fe (22 %)		
	Thin film Au/Fe/Glass					Au-Fe@Au (23 %) + Au-Fe (77 %)		
	Thin film Au/Fe/Au/Fe/Glass					Au-Fe@Au (72 %) + Au-Fe (28 %)		
	Thin film Fe-Au Alloy		1064 nm, 10 ps, 0.08 mJ			Au-Fe@Au (23 %) + Au-Fe (77 %)		
	Thin film Fe/Au/Glass					Au-Fe@Au (41 %) + Au-Fe (59 %)		
	Thin film Au/Fe/Glass					Au-Fe@Au (14 %) + Au-Fe (86 %)		
	Thin film Au/Fe/Au/Fe/Glass					Au-Fe@Au (4 %) + Au-Fe (96 %)		
	Thin film Au/Fe/Glass	Au(50nm)/Fe(120nm)	1064 nm, 10 ps, 0.08 mJ	Ethanol	-	Au-Fe	Au-Fe 81-19 at%	
		Au(50nm)/Fe(70nm)					Au-Fe 80-20 at%	
		Au(95nm)/Fe(56nm)					Au-Fe 89-11 at%	
		Au(175nm)/Fe(56nm)					Au-Fe 93-7 at%	
	Thin film Fe/Au/Glass	Fe(70nm)/Au(50nm)					Au-Fe 60-40 at%	
	Thin film Au/Fe/Au/Fe/Glass	Au(20nm)/Fe(20nm)/ /Au(20nm)/Fe(20nm)					Au-Fe 65-35 at%	
	Thin film Au/Fe/Glass	Au(50nm)/Fe(120nm)		Water		Au-Fe@FeOx	Au-Fe 87-13 at%	
		Au(50nm)/Fe(70nm					Au-Fe 88-12 at%	
		Au(95nm)/Fe(56nm)					Au-Fe 92-8 at%	
		Au(175nm)/Fe(56nm)					Au-Fe 94-6 at%	
	Thin film Fe/Au/Glass	Fe(70nm)/Au(50nm)					Au-Fe 73-27 at%	
	Thin film Au/Fe/Au/Fe/Glass	Au(20nm)/Fe(20nm)/ /Au(20nm)/Fe(20nm)					Au-Fe 87-13 at%	
2 Step LAL	2 distinct targets	Au; Fe	Au: 1025 nm, 420 fs, 50 µJ, 10 kHz<br>Fe: 1025 nm, 420 fs,100 µJ, 10 kHz	H2O	NaCl 1mM	FeOx@Au NPs	FeOx;Au	[84]
LML	Au+Fe3O4 NPs "Large size"	Au NPs 10 to 60 % weight	355 nm, 7 ns, 150 mJ cm^{-2} , 10 Hz	Ethanol	-	Au@Fe3O4 + Au-Fe	Au-Fe from 10-90 to 90-10 wt%	[85]
	Au+Fe3O4 NPs "Small size"					Au-Fe		
	$Au-Fe_3O_4$ NPs	Au NPs 70 to 90 % weight				Au-Fe		
LFL	Fe NPs (wet chemistry)+ commercial Au NPs	Fe44-Au56	532 nm, 7 ns, 65 mJ , 20 Hz	Water/Hexane/Octane	CTAB + N_2 + 1-butanol	Fe@Au	N.D.	[86]

Table S4. Overview of Au-Fe nanostructures obtained with different LPSC techniques. The different structures and compositions obtained with various LSPC techniques in literature are summarized in this table. This work shows the largest number of different atomic structures by using the same laser parameters and target, in addition to only two common solvents as water and ethanol in combination with the three gas-evolving additives. Abbreviations are LSPC: laser synthesis and processing of colloids; LML: laser melting in liquid; LFL: laser fragmentation in liquid.

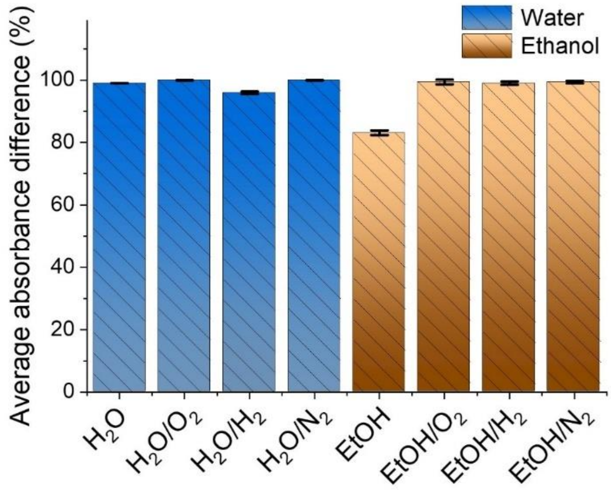

Figure S1. Yield of NPs synthesis after resuspension in pure water. The recovery yield (in %) of the Au-Fe NPs after centrifugation and resuspension in distilled water for the eight samples was calculated from the difference in absorbance of the as-synthesized colloids and the supernatants after centrifugation. The average absorbance difference (in %) at 400, 450, 500 and 550 nm was considered because at these wavelengths there is no overlap with the optical absorption of additives. Error bars represent the standard error among the different wavelengths considered.

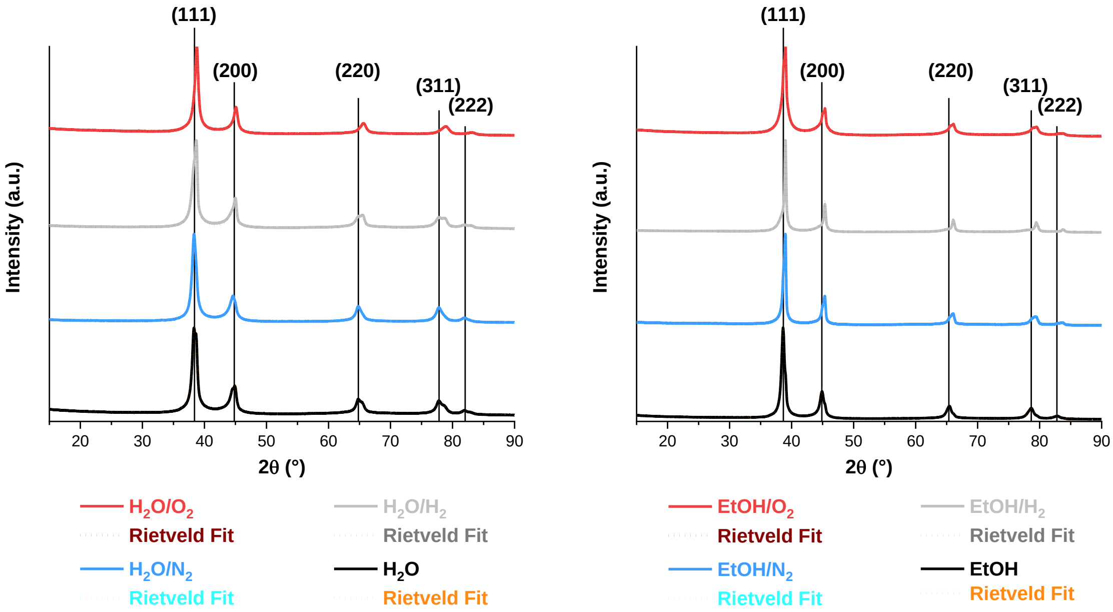

Figure S2. **XRD analysis.** XRD and Rietveld refinement of the eight samples of Au-Fe NPs. The diffractograms were normalized to the most intense reflection of the FCC pattern and vertically shifted for a clearer comparison.

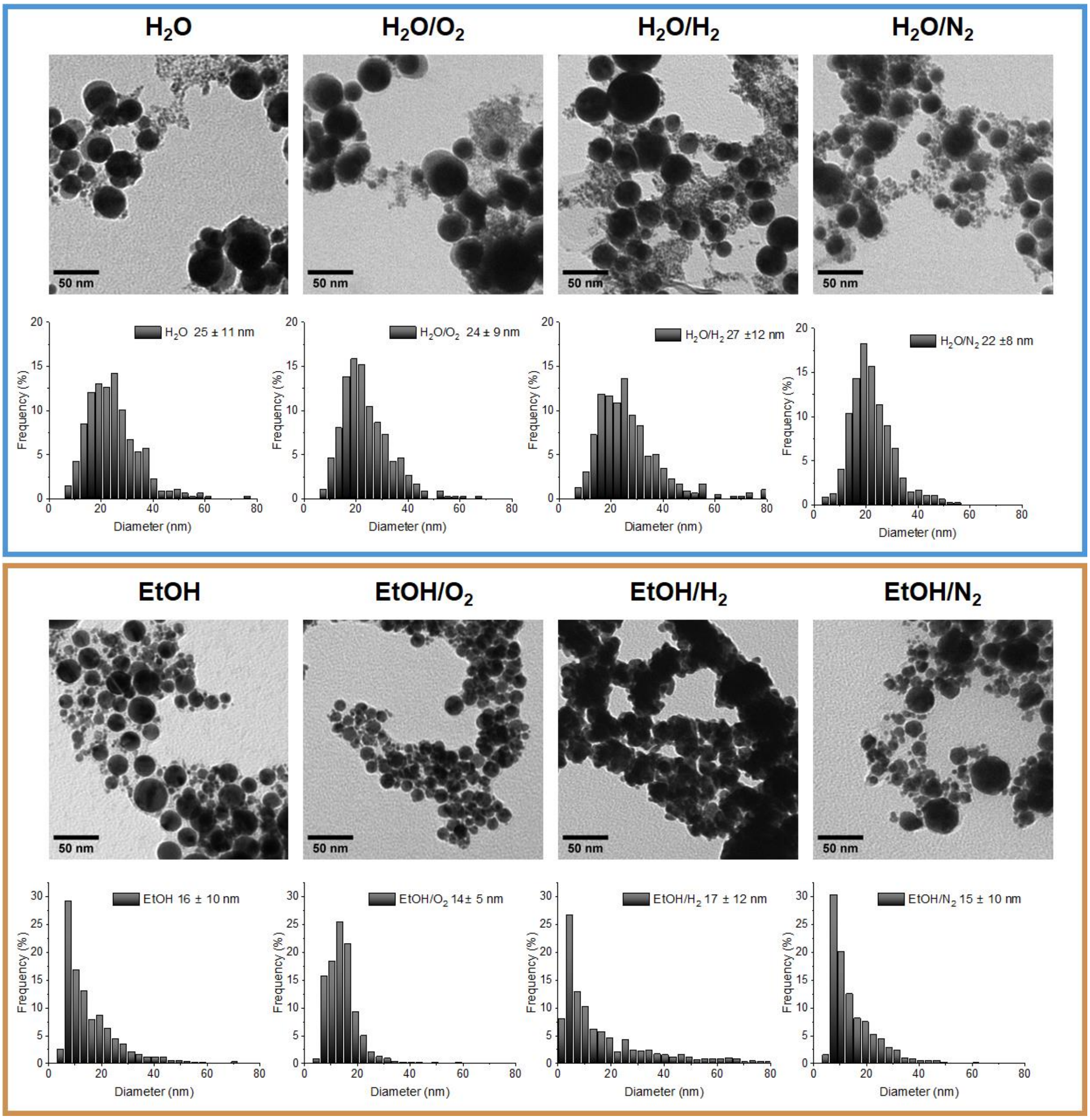

Figure S3. TEM images and histograms. Representative TEM images of the eight samples obtained in distilled water (top) and ethanol (bottom), and the corresponding size histograms. The average size and relative standard deviation, computed on a minimum of 500 NPs, are reported in the histogram legend.

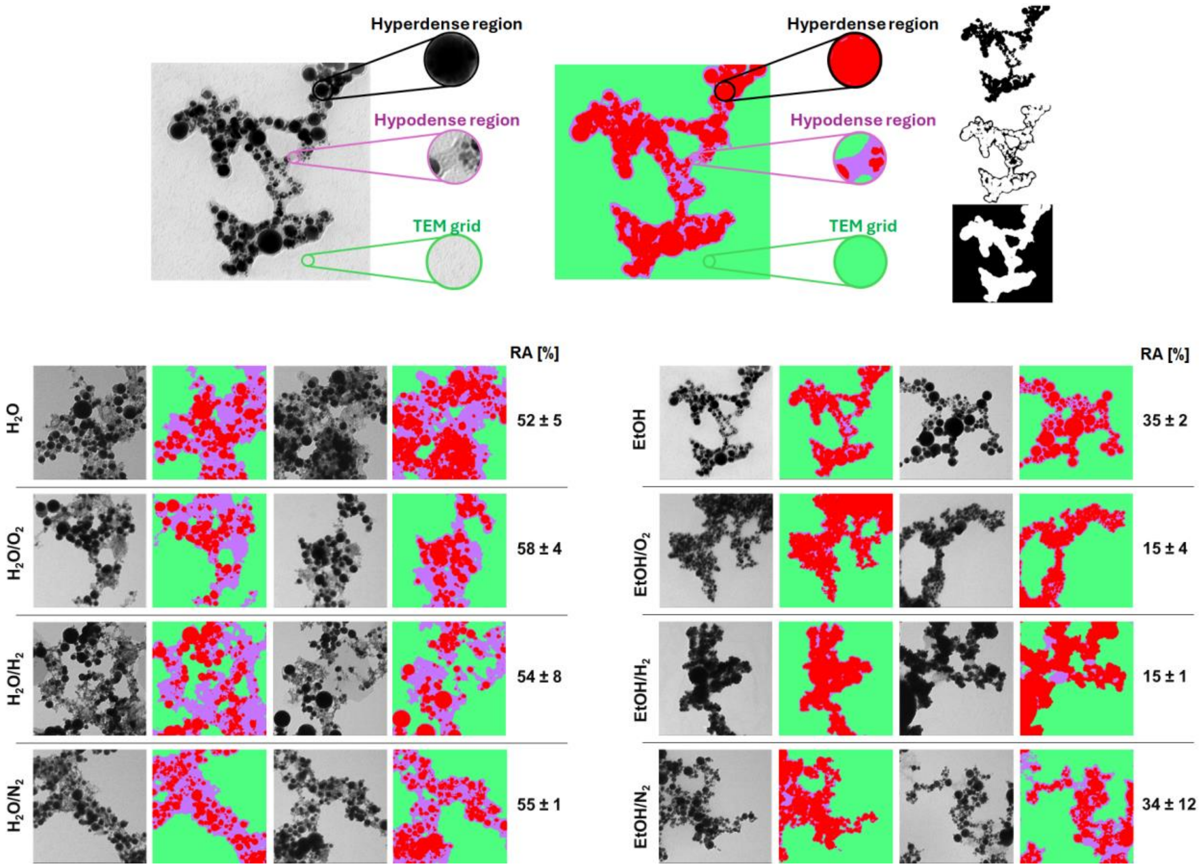

Figure S4. RA calculation. Sketch of the procedure for the calculation of the RA from the identification of hypodense (violet), hyperdense (red) and background (green) regions in the TEM images, and additional TEM images and corresponding segmentation used for the calculation of RA.

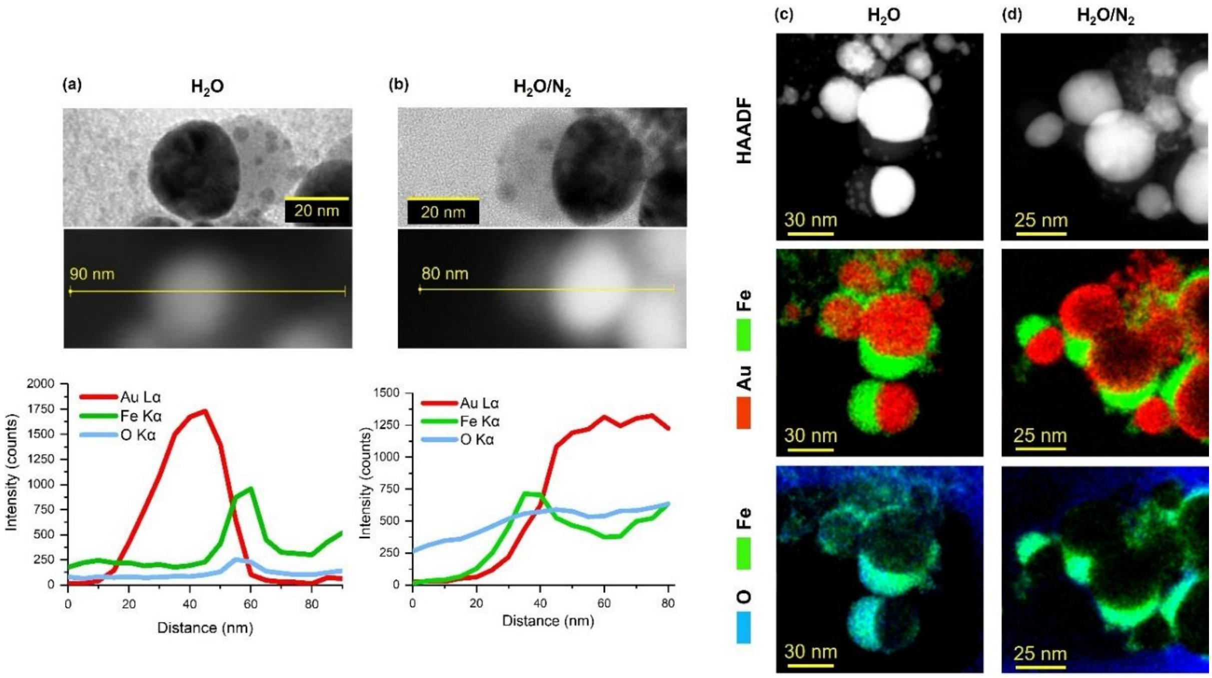

Figure S5. HRTEM and EDX analysis of sample H_2O and H_2O/N_2. (a-b) HRTEM (top), STEM-HAADF (middle) and EDX line profile (bottom) of representative NPs from the H_2O (a) and H_2O/N_2 (b) samples. The Au L (red), Fe K (green) and O K lines were used. (c-d) Additional EDX maps of Au M and Fe K lines (middle), and EDX map of Fe and O K lines (bottom) of representative NPs from the H_2O (c) and H_2O/N_2 (d) samples.

H_2O

(a)

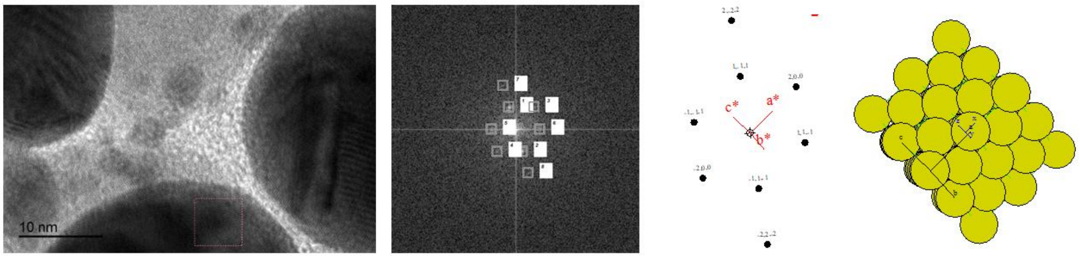

Zone axis [0 1 1]

Spot#	d-Spacing (nm)	Rec. Pos.(1/nm)	Degrees to Spot 1	Degrees to x-axis
1	0.2374	4.212	0	106.84
2	0.2442	4.095	179.39	-73.77
3	0.1921	5.207	55.39	51.45
4	0.1935	5.167	122.57	-130.59
5	0.227	4.405	73.25	-179.91
6	0.2338	4.278	105.87	0.96
7	0.1199	8.342	0.09	106.93
8	0.1225	8.165	179	-74.16

(b)

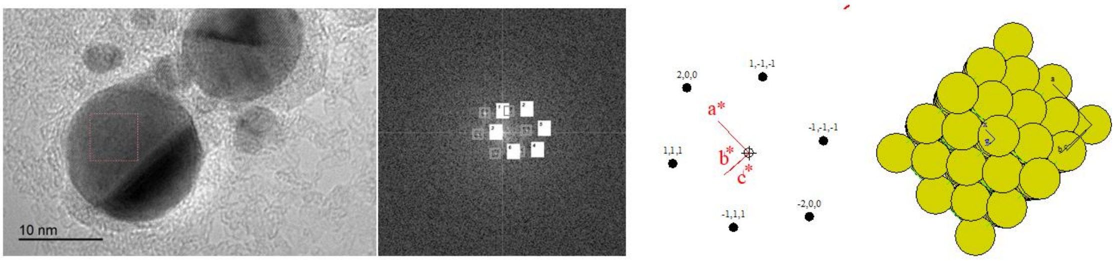

Zone Axis [0 -1 1]

Spot#	d-Spacing (nm)	Rec. Pos.(1/nm)	Degrees to Spot 1	Degrees to x-axis
1	0.2104	4.752	0	132.27
2	0.2469	4.05	58.82	73.45
3	0.2251	4.442	50.98	-176.75
4	0.2152	4.647	179.95	-47.78
5	0.2306	4.336	127.54	4.73
6	0.2522	3.966	119.2	-108.53

(c)

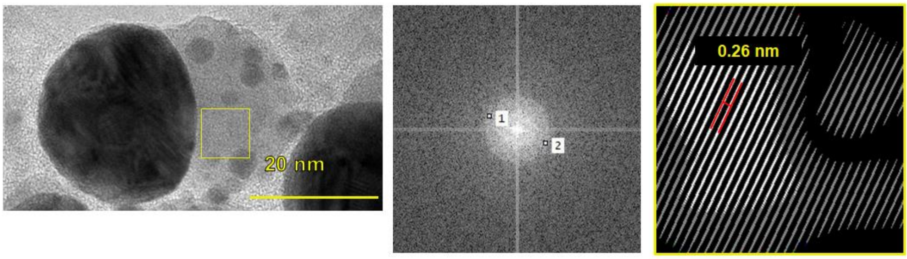

Spot#	d-Spacing (nm)	Rec. Pos.(1/nm)	Degrees to Spot 1	Degrees to x-axis
1	0.2659	3.761	0	154.67
2	0.2607	3.836	179.82	-25.51

Figure S6. HRTEM and FFT analysis of sample H_2O. Representative HRTEM images, associated FFT patterns from the regions delimitated by the red squares in the HRTEM images, and the resulting interplanar distance for NPs in the H_2O sample, matching with FCC Au (hyperdense regions (a) and (b)) and magnetite (hypodense region (c)). Simulated crystal cell in the zone axis [0 1 1] and [0 -1 1] with the corresponding diffraction pattern are also shown (made with the Carine V3.1 software), corresponding to the FFT patterns in (a) and (b).

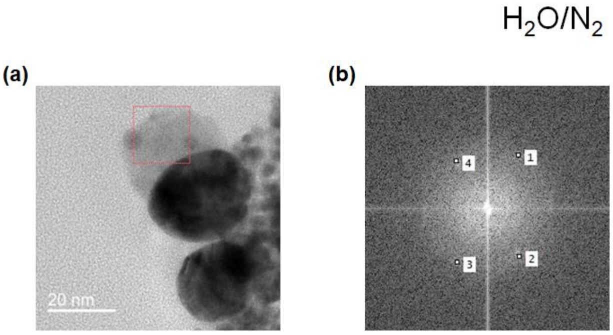

Spot #	d-Spacing (nm)	Rec. Pos.(1/nm)	Degrees to Spot 1	Degrees to x-axis
1	0.2594	3.855	0	56.76
2	0.2767	3.614	116.48	-56.72
3	0.2586	3.868	179.01	-119.25
4	0.2814	3.553	69.39	123.15

Figure S7. HRTEM and FFT analysis of sample H_2O/N_2. Representative HRTEM image, associated FFT patterns from the region delimitated by the red squares in the HRTEM images (a), and the resulting interplanar distance for a NP in the H_2O/N_2 sample (b), matching with magnetite (hypodense region).

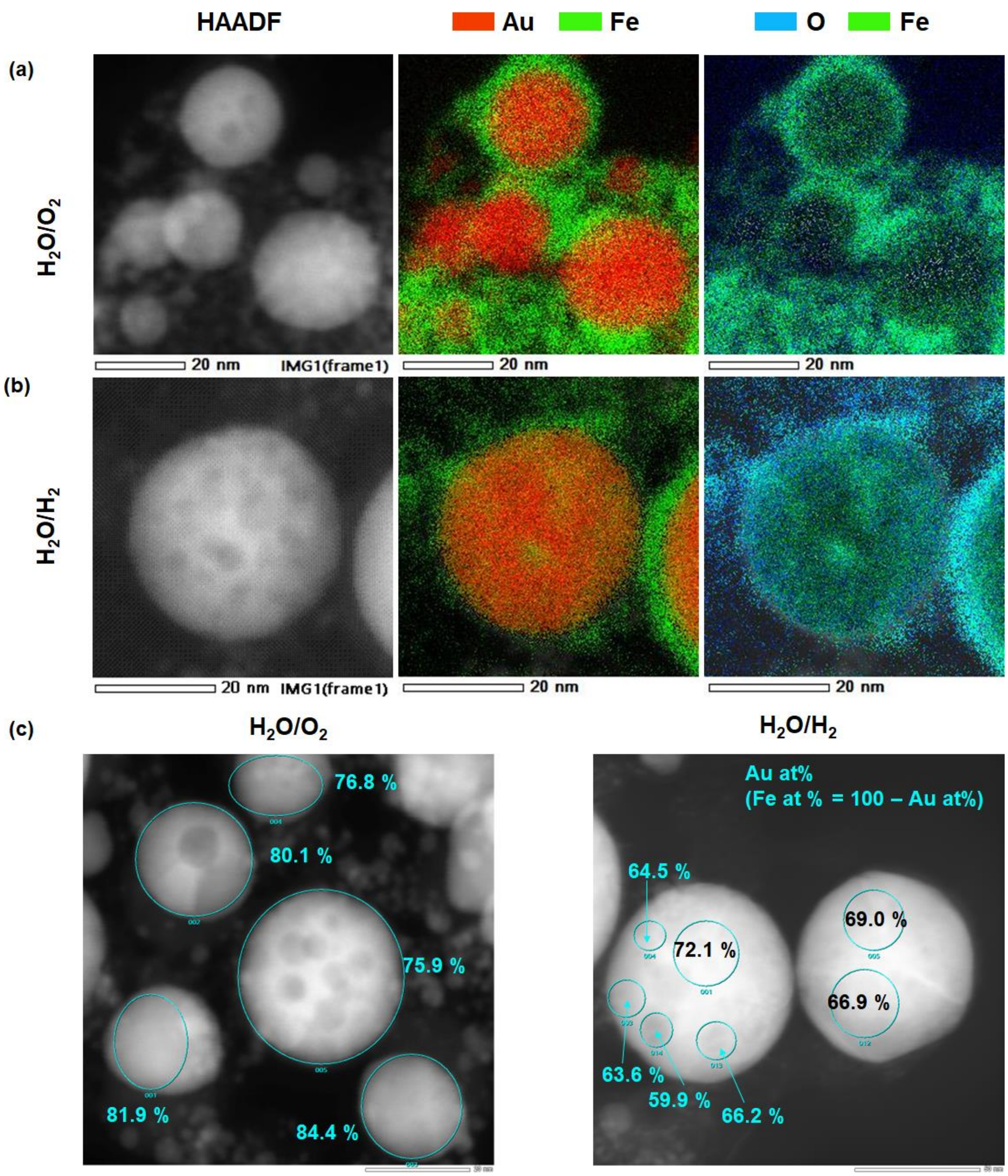

Figure S8. EDX, HRTEM and FFT analysis of sample H_2O/O_2 and H_2O/H_2. Additional STEM-HAADF (left), EDX map of Au M and Fe K lines (center), and EDX map of Fe and O K lines (right) of representative NPs from the H_2O/O_2 (a) and H_2O/H_2 (b) samples. (c) EDX quantitative analysis of Fe at% in various portions of the NPs in the same samples and EDX spectra of hypodense iron-rich precipitates inside the NPs.

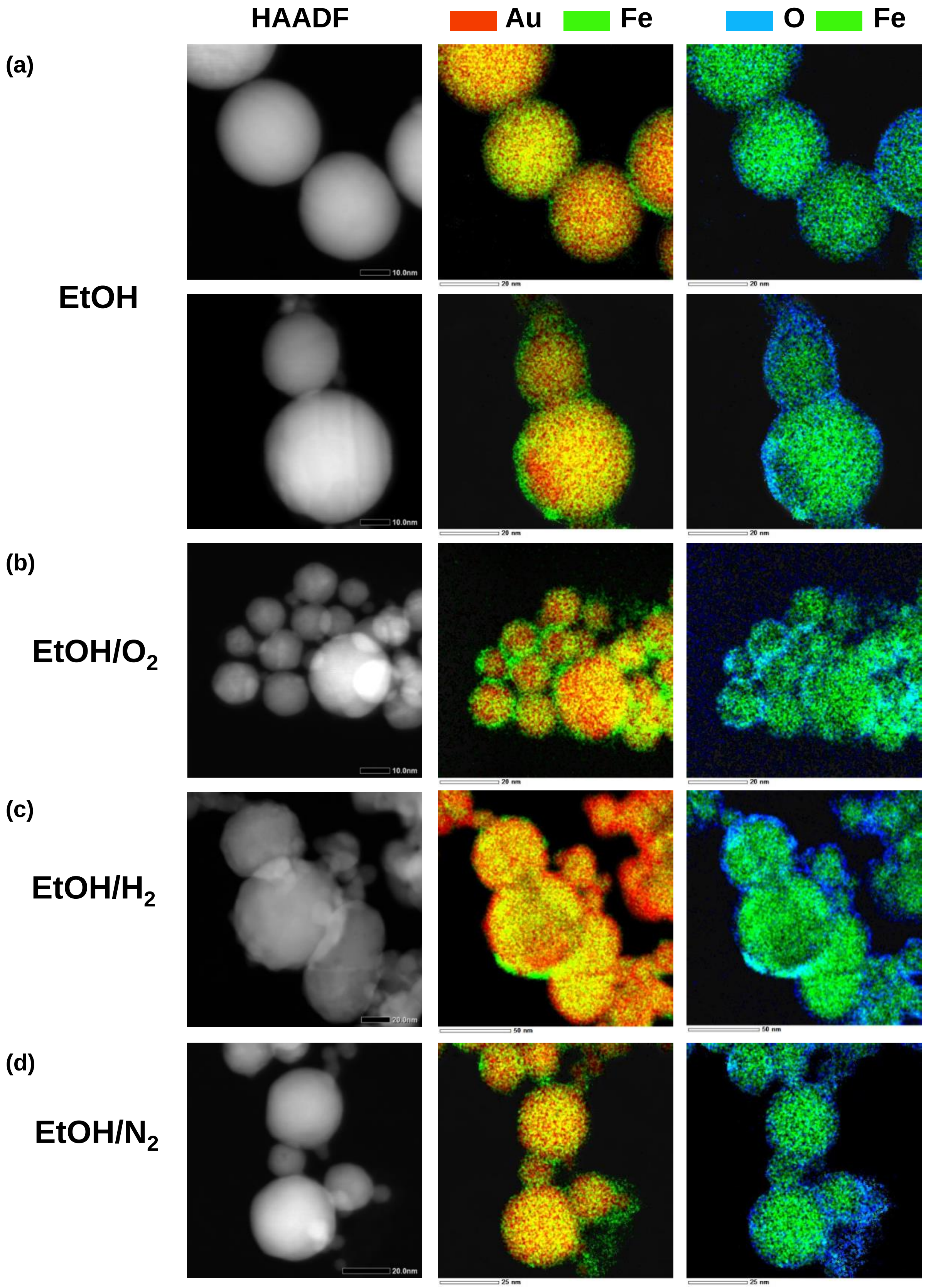

Figure S9. EDX of samples EtOH, EtOH/N$_2$, EtOH/O$_2$ and EtOH/H$_2$. Additional STEM-HAADF (left), EDX map of Au M and Fe K lines (center), and EDX map of Fe and O K lines (right) of representative NPs from the EtOH (a), EtOH/O$_2$ (b), EtOH/H$_2$ (c) and H$_2$O/O$_2$ (d)samples.

EtOH

(a)

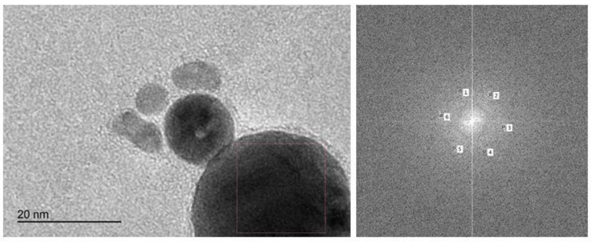

Spot#	d-Spacing (nm)	Rec. Pos.(1/nm)	Degrees to Spot 1	Degrees to x-axis
1	0.2409	4.151	0	112.3
2	0.2402	4.163	56.76	55.44
3	0.2412	4.147	122.32	-10.02
4	0.2387	4.189	179.32	-67.48
5	0.2397	4.172	124.01	-123.69
6	0.2445	4.089	58.36	170.67

(b)

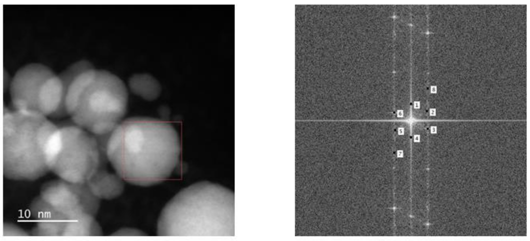

Spot#	d-Spacing (nm)	Rec. Pos.(1/nm)	Degrees to Spot 1	Degrees to x-axis
1	0.2609	3.833	0	90.09
2	0.233	4.293	59.62	30.46
3	0.2312	4.325	115.94	-25.85
4	0.2551	3.920	179.3	-89.21
5	0.2336	4.280	121.01	-148.9
6	0.2348	4.258	64.27	154.35
7	0.119	8.402	152.7	-117.22
8	0.1195	8.369	27.73	62.35

(c)

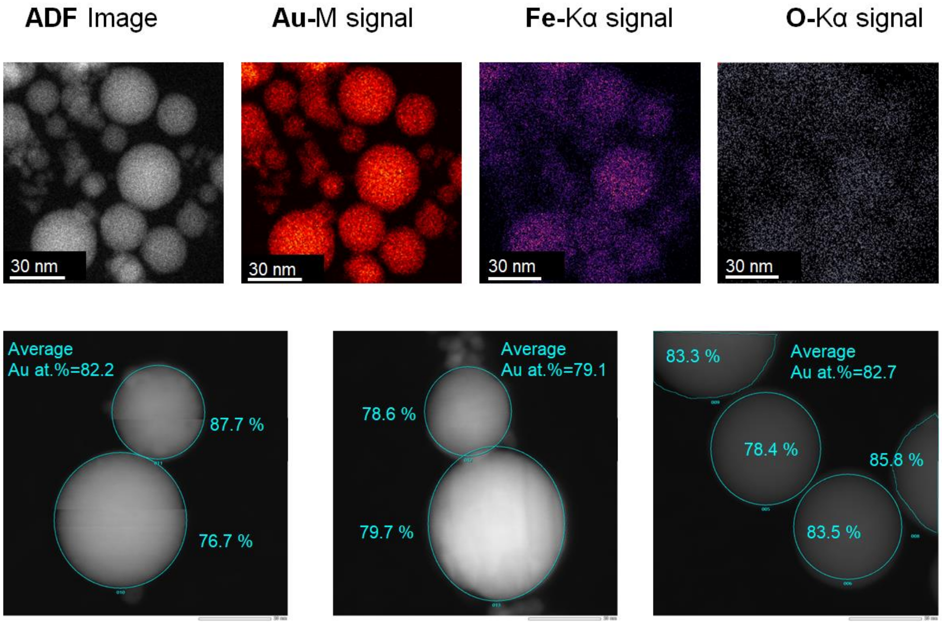

Figure S10. EDX, HRTEM and FFT analysis of samples EtOH. Representative HRTEM (a) and STEM-ADF (b) images, associated FFT patterns from the region delimitated by the red squares in the images, and the resulting interplanar distance for NPs in the EtOH sample, matching with FCC Au. (c) Additional STEM-ADF image and corresponding EDS maps for the Au-M (red), Fe-K (purple) and O-K lines (cyan). (d) EDX quantitative analysis of Fe at% in various portions of the NPs in the same sample.

EtOH/N_2

(a) (b)

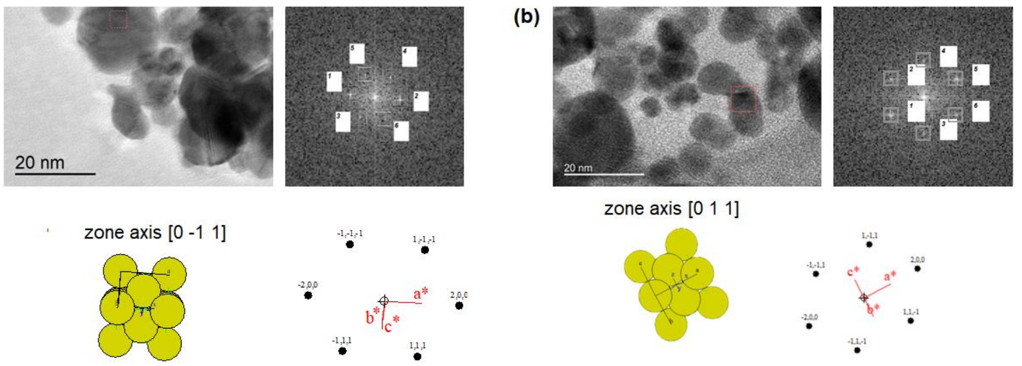

Spot #	hkl	d-Spacing (nm)	Rec. Pos.(1/nm)	Degrees to Spot 1	Degrees to x-axis
1	-2 0 0	0.2358	4.241	0	173.21
2	2 0 0	0.2486	4.023	178.3	-5.09
3	-1 1 1	0.2449	4.083	56.9	-129.89
4	1 -1 -1	0.2463	4.06	119.03	54.18
5	-1 -1 -1	0.24	4.167	58.38	114.83
6	1 1 1	0.2621	3.815	119.42	-67.37

Spot #	hkl	d-Spacing (nm)	Rec. Pos.(1/nm)	Degrees to Spot 1	Degrees to x-axis
1	-2 0 0	0.2411	4.148	0	-151.74
2	2 0 0	0.2404	4.160	57.83	150.43
3	-1 1 -1	0.2540	3.936	60.78	-90.96
4	1 -1 1	0.2456	4.072	117.58	90.69
5	2 0 0	0.2434	4.108	178.58	29.68
6	1 1 -1	0.2488	4.019	122.59	-29.15

(c)

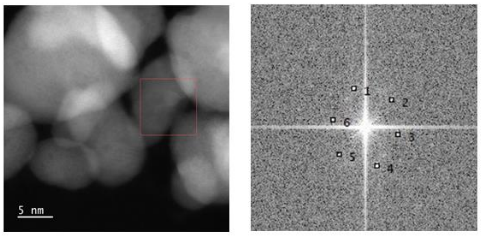

Spot#	d-Spacing (nm)	Rec. Pos.(1/nm)	Degrees to Spot 1	Degrees to x-axis
1	0.2074	4.825	0	106.46
2	0.2225	4.495	60.8	45.66
3	0.2462	4.061	119.86	-13.41
4	0.2046	4.888	179.64	-73.18
5	0.2213	4.520	121.02	-132.52
6	0.2535	3.945	60.99	167.45

(d)

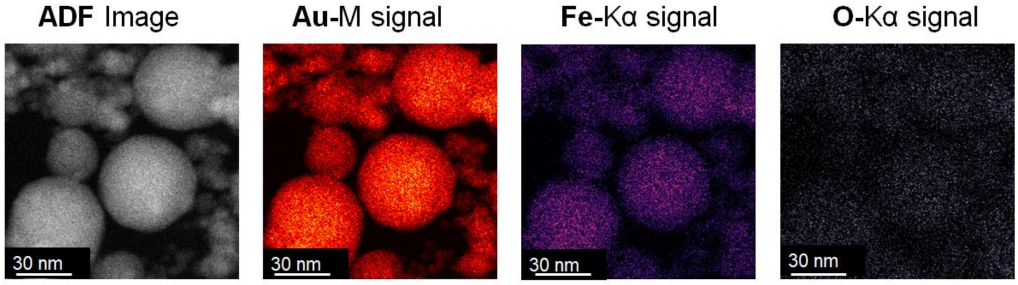

(e)

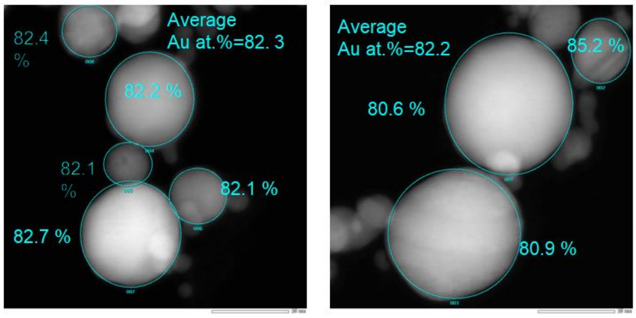

Figure S11. EDX, HRTEM and FFT analysis of samples EtOH/N_2. Representative HRTEM (a-b) and STEM-ADF (c) images, associated FFT patterns from the region delimitated by the red squares in the images, and the resulting interplanar distance for NPs in the EtOH/N_2 sample, matching with FCC Au. Simulated crystal cell in the zone axis [0 -1 1] and [0 1 1] with the corresponding diffraction pattern are also shown (made with the Carine V3.1 software), corresponding to the FFT patterns in (a) and (b). (d) Additional STEM-ADF image and corresponding EDS maps for the Au-M (red), Fe-K (purple) and O-K lines (cyan). (e) EDX quantitative analysis of Fe at% in various portions of the NPs in the same sample.

EtOH/O_2

(a)

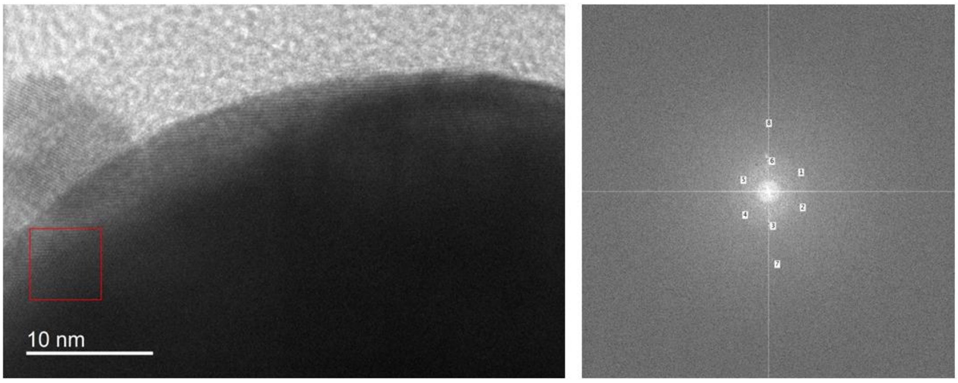

Spot#	d-Spacing (nm)	Rec. Pos.(1/nm)	Degrees to Spot 1	Degrees to x-axis
1	0.2592	3.858	0	90.81
2	0.2648	3.777	179.38	-89.82
3	0.2527	3.957	116.03	-25.22
4	0.247	4.049	63.36	154.16
5	0.2349	4.258	124.56	-144.63
6	0.2358	4.241	54.09	36.71
7	0.1162	8.605	2.95	93.76
8	0.1197	8.354	179.15	-90.04

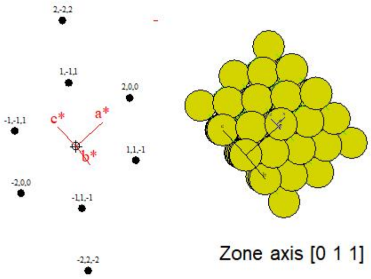

(b)

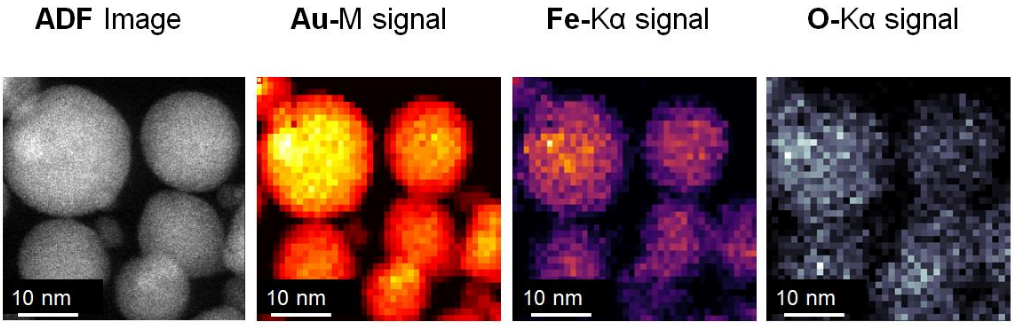

(c)

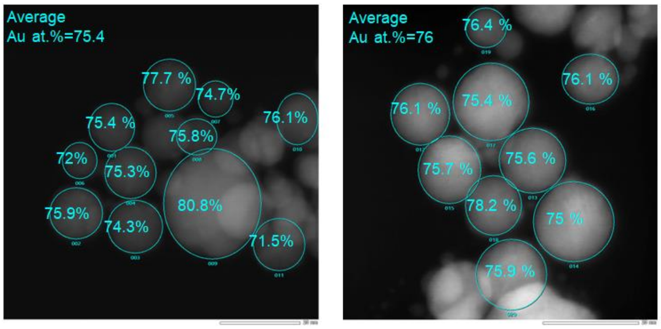

Figure S12. EDX, HRTEM and FFT analysis of samples EtOH/O_2. Representative HRTEM (a) image and the associated FFT pattern from the region delimitated by the red square in the image, and the resulting interplanar distance for NPs in the EtOH/O_2 sample, matching with FCC Au. Simulated crystal cell in the zone axis [0 1 1] with the corresponding diffraction pattern are also shown (made with the Carine V3.1 software), corresponding to the FFT pattern in (a). (b) Additional STEM-ADF image and corresponding EDS maps for the Au-M (red), Fe-K (purple) and O-K lines (cyan). (c) EDX quantitative analysis of Fe at% in various portions of the NPs in the same sample.

EtOH/H_2

(a)

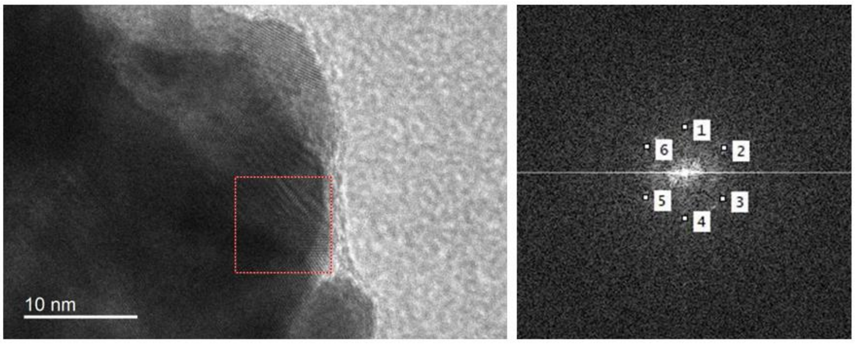

Spot#	d-Spacing (nm)	Rec. Pos.(1/nm)	Degrees to Spot 1	Degrees to x-axis
1	0.2443	4.094	0	89.22
2	0.2393	4.178	57.1	32.11
3	0.2378	4.205	123.9	-34.68
4	0.2412	4.146	179.11	-89.9
5	0.2397	4.171	124.2	-146.59
6	0.2425	4.123	56.41	145.62

(b)

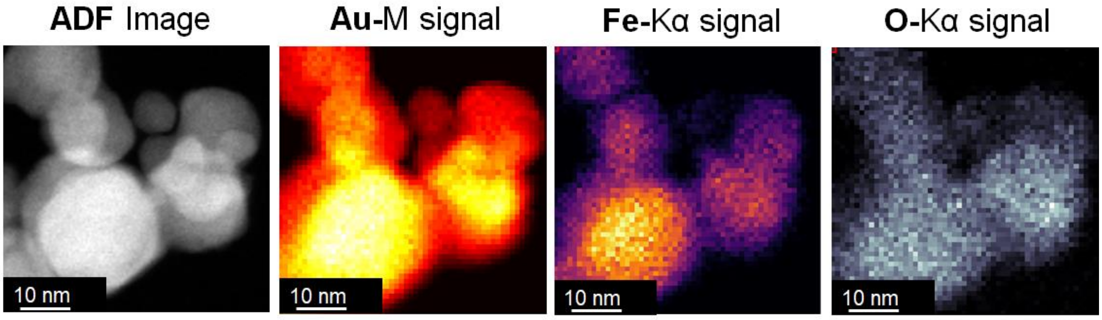

(c)

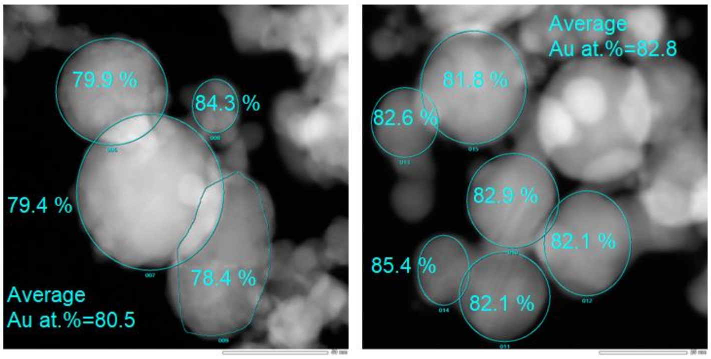

Figure S13. EDX, HRTEM and FFT analysis of samples EtOH/H_2. Representative HRTEM (a) image and the associated FFT pattern from the region delimitated by the red square in the image, and the resulting interplanar distance for NPs in the EtOH/H_2 sample, matching with FCC. (b) Additional STEM-ADF image and corresponding EDS maps for the Au-M (red), Fe-K (purple) and O-K lines (cyan). (c) EDX quantitative analysis of Fe at% in various portions of the NPs in the same sample.

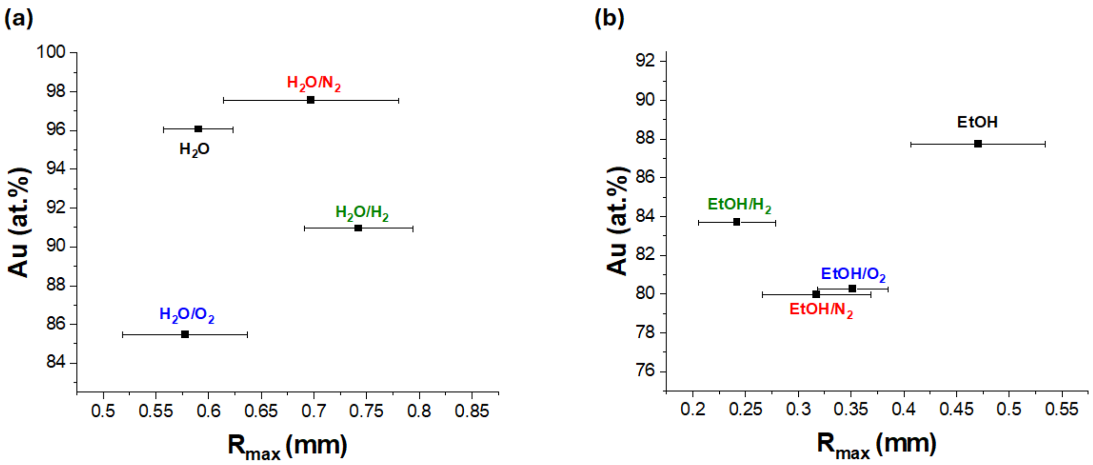

Figure S14. Plot of R_{MAX} versus composition. Plot of the Au at% obtained from XRD versus the cavitation bubble R_{MAX} in water (a) and ethanol (b) samples. No correlation between the Au content and bubble maximum size is observed.

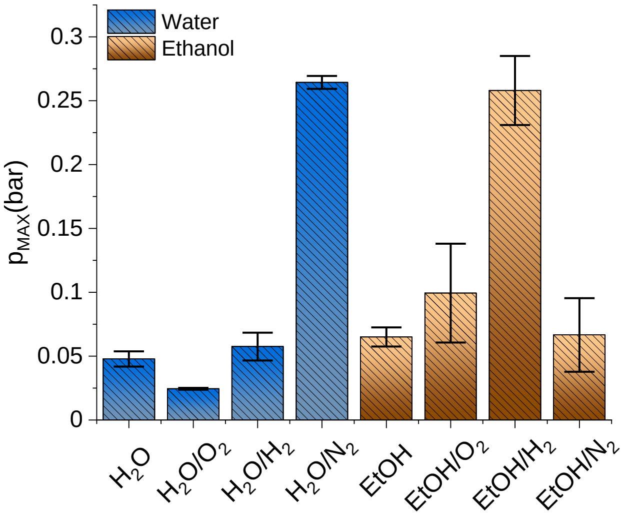

Figure S15. Cavitation bubble pressure at R_{MAX}. Calculated bubble pressure at the maximum radius (p_{MAX}) for the eight samples in water and ethanol.

4. Thermodynamic model for Gibbs free energy in Au-Fe NPs.[75,81,87–89]

The Gibbs free energy (G) was separately calculated for solid solution (SS) and core-shell (CS) nanoparticles. In the SS there is a homogeneous distribution of both elements within the particle, while the CS is composed of an iron core covered by a gold shell, namely the hypothesis of complete element segregation is applied. The thermodynamic model considers spherical particles with no faceting according to the experimental evidence derived from TEM analysis. Therefore, the average surface energy values are used, namely differences in interface/surface energies for different facets are ignored. It is also assumed that there is no stress at the interface and interactions with the solvent are not considered in this model, according to analogous studies reported in literature.[75,81,87–89]

1. Model for calculation of the Gibbs free energy for SS NPs (G_{SS})

$$G_{ss} = X_{Au}G_{Au} + X_{Fe}G_{Fe} + \Delta G_{mix} + (2\gamma_{ss}V_{AuFe})/(D/2) \quad \text{(Equation S9)}$$

with

D: the diameter of the particle

X_{Fe} , X_{Au} : the mole fraction of Au and Fe respectively

G_{Au} , G_{Fe} : the molar free energies of pure Au (G_{Au} = -14.1kJ/mol) and Fe (G_{Fe} = -7.95 kJ/mol)[90]

$\Delta G_{mix} = \Delta H_{mix} - T\Delta S_{mix}$: the excess free energy of the alloy phase due to mixing (ΔG_{mix} = 39.296 kJ/ mol)[91]

γ_{ss} : the size dependent specific surface energy (surface energy per unit area) of the nanoparticle with solid solution of Au and Fe atoms.

2. Model for calculation of the Gibbs free energy for CS NPs (G_{CS})

$$G_{CS} = X_{Au}G_{Au} + X_{Fe}G_{Fe} + \alpha_{Au}S_{Au}\gamma_{Au} + \alpha_{Fe}S_{Fe}\gamma_{Fe} + G_{int} \quad \text{(Equation S10)}$$

with

X_{Fe} , X_{Au} : the mole fraction of Au and Fe respectively

G_{Au} , G_{Fe} : the molar free energies of pure Au (G_{Au} = -14.1kJ/mol) and Fe (G_{Fe} = -7.95 kJ/mol)[90]

α_{Au} , α_{Fe} : the fractions of surface atoms of Au and Fe versus the total number of Au and Fe atoms in the particle

S_{Au} , S_{Fe}: the surface areas occupied by 1 mole of atoms of Au and Fe, respectively

γ_{Au} , γ_{Fe} : the size dependent specific surface energy (surface energy per unit area) of the nanoparticle for either Au and Fe atoms

G_{int} : the Gibbs free energy contribution given by the geometrical (**γ_{geo}**) and chemical (**γ_{chem}**) factors.

Additional details on the calculation of G_{SS} and G_{CS} are reported in ref.[75] and the other ref.[81,87–89].

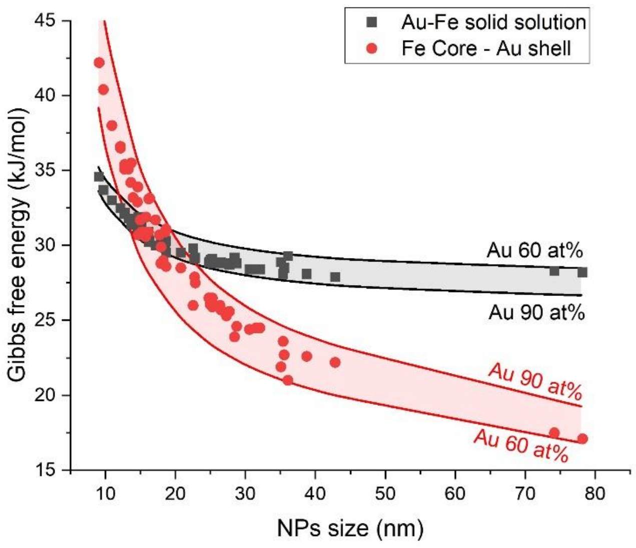

Figure S16. Plot of Gibbs free energy for Au-Fe NPs with SS or CS morphology. The Gibbs free energy is calculated for Au-Fe NPs of different size and composition considering the solid solution (black lines, top line: Au 60 at %; bottom line: Au 90 at%) and the core-shell (Fe core, Au shell, red lines, top line: Au 90 at %; bottom line: Au 60 at%) morphologies. The calculations are performed for NPs in vacuum. Each point (black square for SS, red circles for CS) corresponds to one an Au-Fe NPs analyzed by EDX in this study.

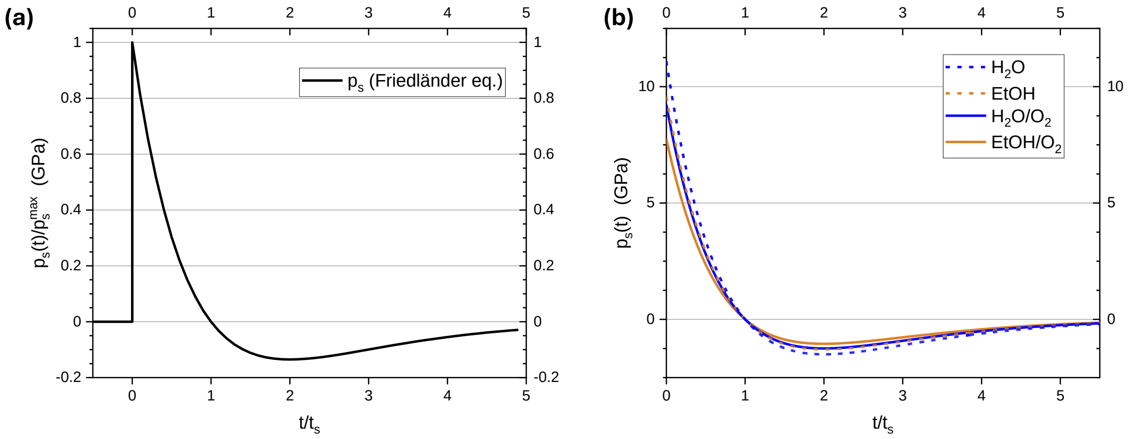

Figure S17. Plot of shock wave pressure with the Friedländer equation. (a) The normalized overpressure $p_s(t)/p_s^{max}$ as a function of the normalized delay t/t_s from Friedländer equation with $\alpha = 1$. (b) Comparison of the $p_s(t)$ for H_2O, H_2O/O_2, EtOH, EtOH/O_2 as a function of the normalized delay t/t_s under the hypothesis that Friedländer equation with $\alpha = 1$ can be applied to the experimental case. Note that the Friedländer equation applies to open environments only, but numerical simulations and direct measurements showed similar behaviour in liquids, with the same order of magnitude in the ratio between the maximum overpressure and the negative pressure.

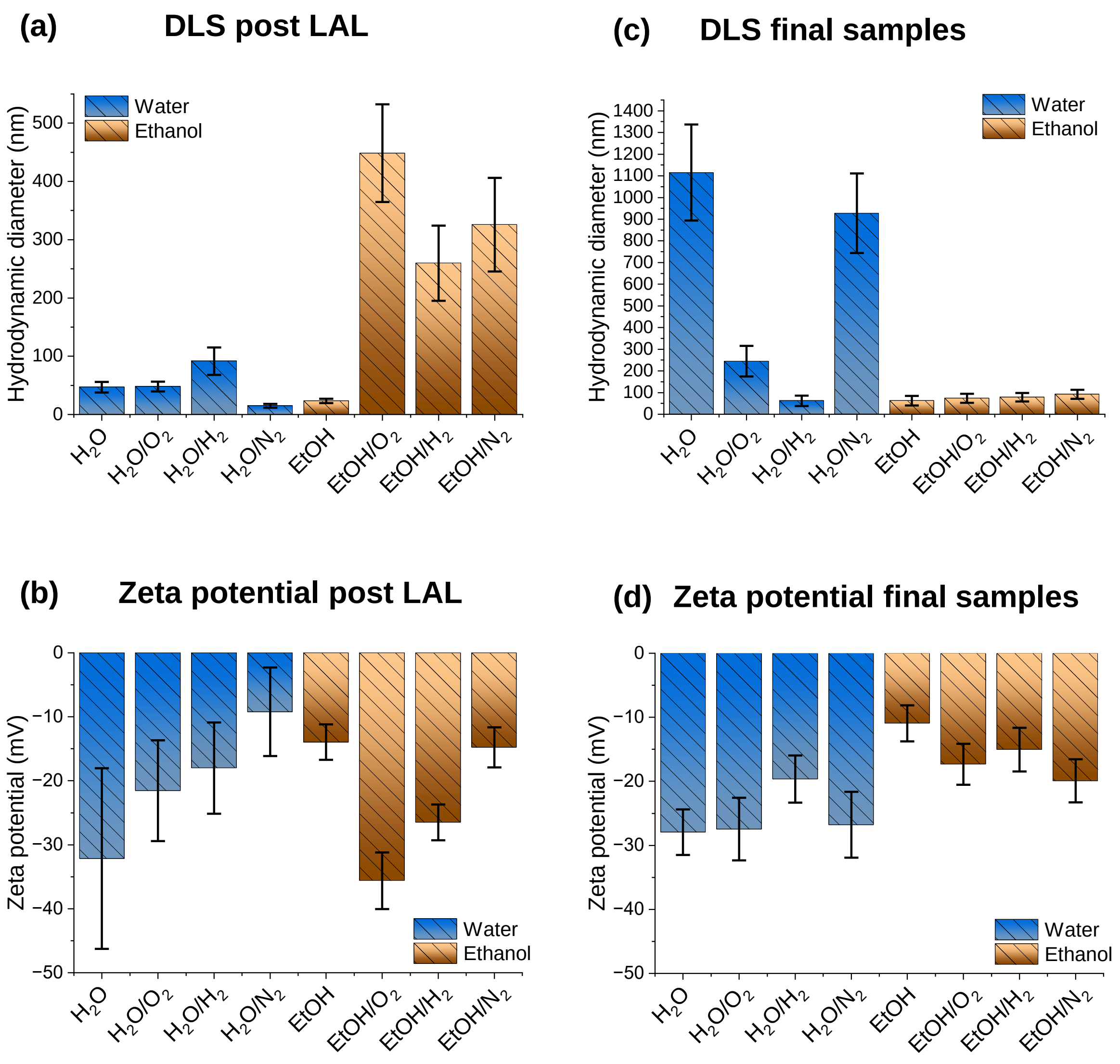

Figure S18. DLS and z-potential of Au-Fe samples. The hydrodynamic size and the z-potential are measured on the as-synthesized colloids (a-b) and on the final samples resuspended in water (c-d). The results are reported as the average of three measurements with the relative standard error.

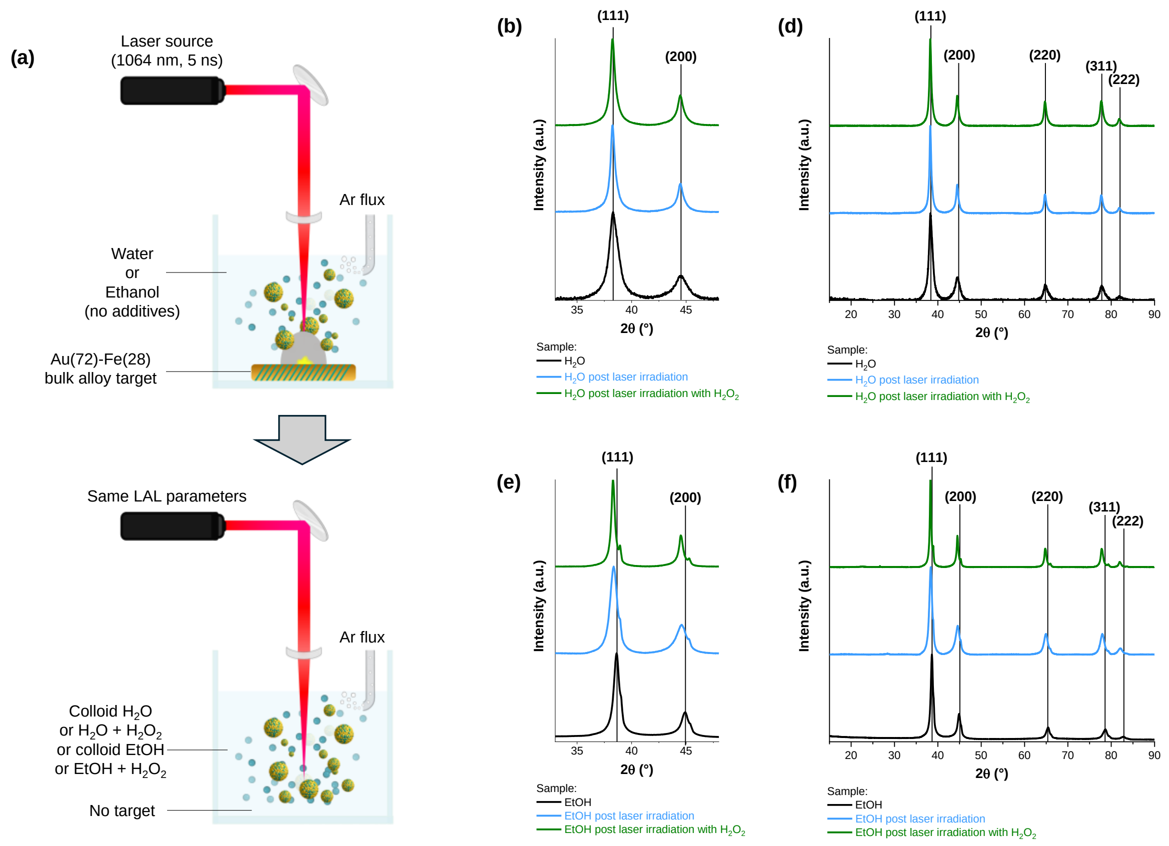

Figure S19. Effect of reirradiation on Au-Fe samples. The effect of reirradiation was tested on the Au-Fe samples obtained in water (H_2O sample) and ethanol (EtOH sample). The as-synthesized colloid was subjected to another LAL cycle after removing the target. The experiment was performed on the colloid in the pure solvents (either water or ethanol) or after the addition of H_2O_2 to verify the effect of this additive during reirradiation, as sketched in (a). (b-f) The XRD patterns of the NPs samples were registered. In samples in water, no re-alloying was observed according to the position of the FCC (111) and (200) peaks compared to the not-irradiated NPs (b, c). In ethanol, the shift of the diffraction peaks to smaller angles (larger lattice parameters) compared to the not-irradiated NPs indicates that reirradiation has the effect of dealloying. The effect is the same also when H_2O_2 is present, hence reirradiation of NPs generated by LAL in H_2O/O_2 and $EtOH/O_2$ samples does not account for the observed structural differences (higher yield of Au-Fe substitutional alloys). Vertical lines identify the position of diffraction peaks in the not-irradiated samples.

References

[1] V. Amendola, M. Meneghetti, O. M. Bakr, P. Riello, S. Polizzi, D. H. Anjum, S. Fiameni, P. Arosio, T. Orlando, C. de Julian Fernandez, F. Pineider, C. Sangregorio, A. Lascialfari, H. Dalaver, P. Arosio, T. Orlando, C. de Julian Fernandez, F. Pineider, C. Sangregorio, A. Lascialfari, Coexistence of Plasmonic and Magnetic Properties in Au89Fe11 Nanoalloys, *Nanoscale* **2013**, *5*, 5611.

[2] T. Wagner, J. Eglinger, Thorstenwagner/Ij-Particlesizer: V1.0.9 Snapshot Release. **2017**. https://doi.org/https://zenodo.org/badge/DOI/10.5281/zenodo.820296.svg.

[3] I. Arganda-Carreras, V. Kaynig, C. Rueden, K. W. Eliceiri, J. Schindelin, A. Cardona, H. Sebastian Seung, Trainable Weka Segmentation: A Machine Learning Tool for Microscopy Pixel Classification, *Bioinformatics* **2017**, *33*, 2424.

[4] I. Martial, F. Balembois, J. Didierjean, P. Georges, Nd:YAG Single-Crystal Fiber as High Peak Power Amplifier of Pulses below One Nanosecond, *Opt. Express* **2011**, *19*, 11667.

[5] J. Lam, J. Lombard, C. Dujardin, G. Ledoux, S. Merabia, D. Amans, Dynamical Study of Bubble Expansion Following Laser Ablation in Liquids, *Appl. Phys. Lett.* **2016**, *108*, 074104.

[6] J. M. Walsh, M. H. Rice, R. G. McQueen, F. L. Yarger, Shock-Wave Compressions of Twenty-Seven Metals. Equations of State of Metals, *Phys. Rev.* **1957**, *108*, 196.

[7] G. ~E. Duvall, G. ~R. Fowles, in *High Pressure Physics and Chemistry, Volume 2* (Ed.: R. ~S. Bradley), Vol. 2, **1963**, p. 209.

[8] R. W. Woolfolk, M. Cowperthwaite, R. Shaw, "Universal" Hugoniot for Liquids, *Thermochim. Acta* **1973**, *5*, 409.

[9] M. H. Rice, J. M. Walsh, Equation of State of Water to 250 Kilobars, *J. Chem. Phys.* **1957**, *26*, 824.

[10] T. Tsuneda, T. Taketsugu, Theoretical Investigations on Hydrogen Peroxide Decomposition in Aquo, *Phys. Chem. Chem. Phys.* **2018**, *20*, 24992.

[11] C. K. Mclane, Thermal Decomposition of Hydrogen Peroxide, *J. Chem. Phys.* **1949**, 17, 379

[12] C. Jubert Tomasso, A. L. Pham, T. M. Mattox, J. J. Urban, Using Additives to Control the Decomposition Temperature of Sodium Borohydride, *J. Energy Power Technol. 2020, Vol. 2, 009* **2020**, *2*, 1.

[13] S. Shin, Y. Kim, J. H. Jin, J. Jung, Heat-Induced Dry Hydrolysis of Sodium Borohydride/Oxalic Acid Dihydrate Composite for Hydrogen Production, *ACS Omega* **2022**, *7*, 979.

[14] E. Welchman, T. Thonhauser, Decomposition Mechanisms in Metal Borohydrides and Their Ammoniates, *J. Mater. Chem. A* **2017**, *5*, 4084.

[15] H. Potvin, M. H. Back, A Study of the Decomposition of Sodium Azide Using Differential Thermal Analysis, *Can. J. Chem.* **1973**, *51*, 183.

[16] E. M. Bulewicz, C. G. James, T. M. Sugden, Thermochemistry and Reactivity of the Azides - I. Thermochemistry of the Inorganic Azides, *Proc. R. Soc. London. Ser. A. Math. Phys. Sci.* **1956**, *235*, 106.

[17] E. A. Betterton, *Environmental Fate of Sodium Azide Derived from Automobile Airbags*, Vol. 33, Taylor & Francis **2003**.

[18] H. Nakamura, K. Sakumoto, Y. Hara, K. Ochi, Thermal Analysis of Sodium Azide, *J.*

Hazard. Mater. **1994**, *38*, 1.

[19] J. Reguera, D. Jiménez de Aberasturi, M. Henriksen-Lacey, J. Langer, A. Espinosa, B. Szczupak, C. Wilhelm, L. M. Liz-Marzán, Janus Plasmonic–Magnetic Gold–Iron Oxide Nanoparticles as Contrast Agents for Multimodal Imaging, *Nanoscale* **2017**, *9*, 9467.

[20] J. Song, B. Wu, Z. Zhou, G. Zhu, Y. Liu, Z. Yang, L. Lin, G. Yu, F. Zhang, G. Zhang, H. Duan, G. D. Stucky, X. Chen, Double-Layered Plasmonic–Magnetic Vesicles by Self-Assembly of Janus Amphiphilic Gold–Iron(II,III) Oxide Nanoparticles, *Angew. Chemie Int. Ed.* **2017**, *56*, 8110.

[21] C. Wang, Y. Yao, Q. Song, Gold Nanoclusters Decorated with Magnetic Iron Oxide Nanoparticles for Potential Multimodal Optical/Magnetic Resonance Imaging, *J. Mater. Chem. C* **2015**, *3*, 5910.

[22] X. Chen, G. Li, Q. Han, X. Li, L. Li, T. Wang, C. Wang, Rational Design of Branched Au–Fe 3 O 4 Janus Nanoparticles for Simultaneous Trimodal Imaging and Photothermal Therapy of Cancer Cells. Chem, *Chem. – A Eur. J.* **2017**, *23*, 17204.

[23] R. Wei, Z. Li, B. Kang, G. Fu, K. Zhang, M. Xue, Acid-Triggered in Vivo Aggregation of Janus Nanoparticles for Enhanced Imaging-Guided Photothermal Therapy, *Nanoscale Adv.* **2023**, *5*, 268.

[24] S. Park, J. Choi, N. Ko, S. Mondal, U. Pal, B.-I. Lee, J. Oh, Beta Cyclodextrin Conjugated Au Fe3O4 Janus Nanoparticles with Enhanced Chemo-Photothermal Therapy Performance, *Acta Biomater.* **2024**, *182*, 213.

[25] N. Kostevsek, E. Locatelli, C. Garrovo, F. Arena, I. Monaco, I. P. Nikolov, S. Sturm, K. Zuzek Rozman, V. Lorusso, P. Giustetto, P. Bardini, S. Biffi, M. Comes Franchini, Surface Functionalization of Dumbbell-like Gold–Iron Oxide Nanoparticles: A Chitosan-Based Nanotheranostic System, *Chem. Commun.* **2016**, *52*, 378.

[26] L. León Félix, B. Sanz, V. Sebastián, T. E. Torres, M. H. Sousa, J. A. H. Coaquira, M. R. Ibarra, G. F. Goya, Gold-Decorated Magnetic Nanoparticles Design for Hyperthermia Applications and as a Potential Platform for Their Surface-Functionalization, *Sci. Rep.* **2019**, *9*, 4185.

[27] A. Espinosa, J. Reguera, A. Curcio, Á. Muñoz-Noval, C. Kuttner, A. Van de Walle, L. M. Liz-Marzán, C. Wilhelm, Janus Magnetic-Plasmonic Nanoparticles for Magnetically Guided and Thermally Activated Cancer Therapy, *Small* **2020**, *16*.

[28] E. S. Abu Serea, I. Orue, J. Á. García, S. Lanceros-Méndez, J. Reguera, Enhancement and Tunability of Plasmonic-Magnetic Hyperthermia through Shape and Size Control of Au:Fe 3 O 4 Janus Nanoparticles, *ACS Appl. Nano Mater.* **2023**, *6*, 18466.

[29] R. Wei, G. Fu, Z. Li, Y. Liu, L. Qi, K. Liu, Z. Zhao, M. Xue, Janus Nanoparticles for Imaging-Guided near Infrared-Enhanced Ferroptosis Therapy in Triple Negative Breast Cancer, *J. Colloid Interface Sci.* **2024**, *663*, 644.

[30] T. Chen, J. Yang, H. Zhao, D. Li, X. Luo, Z. Fan, B. Ren, Y. Cai, R. Dong, Ultrasound-Propelled Nanomotors for Efficient Cancer Cell Ferroptosis, *J. Mater. Chem. B* **2024**, *12*, 667.

[31] H. Nosrati, Y. Baghdadchi, R. Abbasi, M. Barsbay, M. Ghaffarlou, F. Abhari, A. Mohammadi, T. Kavetskyy, S. Bochani, H. Rezaeejam, S. Davaran, H. Danafar, Iron Oxide and Gold Bimetallic Radiosensitizers for Synchronous Tumor Chemoradiation Therapy in 4T1 Breast Cancer Murine Model, *J. Mater. Chem. B* **2021**, *9*, 4510.

[32] J. R. Hwu, Y. S. Lin, T. Josephrajan, M.-H. Hsu, F.-Y. Cheng, C.-S. Yeh, W.-C. Su, D.-

B. Shieh, Targeted Paclitaxel by Conjugation to Iron Oxide and Gold Nanoparticles, *J. Am. Chem. Soc.* **2009**, *131*, 66.

[33] H. Kakwere, M. E. Materia, A. Curcio, M. Prato, A. Sathya, S. Nitti, T. Pellegrino, Dually Responsive Gold–Iron Oxide Heterodimers: Merging Stimuli-Responsive Surface Properties with Intrinsic Inorganic Material Features, *Nanoscale* **2018**, *10*, 3930.

[34] M. Baneshi, S. Dadfarnia, A. M. H. Shabani, S. K. Sabbagh, S. Haghgoo, H. Bardania, A Novel Theranostic System of AS1411 Aptamer-Functionalized Albumin nanoparticles Loaded on Iron Oxide and Gold Nanoparticles for Doxorubicin Delivery, *Int. J. Pharm.* **2019**, *564*, 145.

[35] L. Landgraf, P. Ernst, I. Schick, O. Köhler, H. Oehring, W. Tremel, I. Hilger, Anti-Oxidative Effects and Harmlessness of Asymmetric Au@Fe3O4 Janus Particles on Human Blood Cells, *Biomaterials* **2014**, *35*, 6986.

[36] T. Kinoshita, S. Seino, Y. Mizukoshi, Y. Otome, T. Nakagawa, K. Okitsu, T. A. Yamamoto, Magnetic Separation of Amino Acids by Gold/Iron-Oxide Composite Nanoparticles Synthesized by Gamma-Ray Irradiation, *J. Magn. Magn. Mater.* **2005**, *293*, 106.

[37] J. S. Aaron, J. Oh, T. A. Larson, S. Kumar, T. E. Milner, K. V. Sokolov, Increased Optical Contrast in Imaging of Epidermal Growth Factor Receptor Using Magnetically Actuated Hybrid Gold/Iron Oxide Nanoparticles, *Opt. Express* **2006**, *14*, 12930.

[38] Y. Dong, C. Wen, Y. She, Y. Zhang, Y. Chen, J. Zeng, Magnetic Relaxation Switching Immunoassay Based on Hydrogen Peroxide-Mediated Assembly of Ag@Au–Fe 3 O 4 Nanoprobe for Detection of Aflatoxin B1, *Small* **2021**, *17*.

[39] A. Kumar, B. Purohit, K. Mahato, R. Mandal, A. Srivastava, P. Chandra, Gold-Iron Bimetallic Nanoparticles Impregnated Reduced Graphene Oxide Based Nanosensor for Label-free Detection of Biomarker Related to Non-alcoholic Fatty Liver Disease, *Electroanalysis* **2019**, *31*, 2417.

[40] Y. Teng, J. Shi, P. W. T. Pong, Sensitive and Specific Colorimetric Detection of Cancer Cells Based on Folate-Conjugated Gold–Iron-Oxide Composite Nanoparticles, *ACS Appl. Nano Mater.* **2019**, *2*, 7421.

[41] S. Yu, D. Zhang, Y. Zhang, W. Pan, B. E. Meteku, F. Zhang, J. Zeng, Green Light-Driven Enhanced Ammonia Sensing at Room Temperature Based on Seed-Mediated Growth of Gold-Ferrosoferric Oxide Dumbbell-like Heteronanostructures, *Nanoscale* **2020**, *12*, 18815.

[42] Y. Zhang, M. Gong, X. Li, H. Liu, P. Liang, S. Cui, L. Zhang, C. Zhou, T. Sun, M. Zhang, C.-Y. Wen, J. Zeng, Au-Fe3O4 Heterodimer Multifunctional Nanoparticles-Based Platform for Ultrasensitive Naked-Eye Detection of Salmonella Typhimurium, *J. Hazard. Mater.* **2022**, *436*, 129140.

[43] F. U. Haq, A. Batool, S. Niazi, I. M. Khan, A. Raza, D. Ning, Y. Zhang, Z. Wang, Development of Novel FRET Aptasensor Based on the Quenching Ability of Iron Oxide-Gold Nanostars for the Detection of Aflatoxin M1, *Food Chem.* **2025**, *464*, 141575.

[44] X. F. Zhang, L. Clime, H. Q. Ly, M. Trudeau, T. Veres, Multifunctional Fe 3 O 4 −Au/Porous Silica@Fluorescein Core/Shell Nanoparticles with Enhanced Fluorescence Quantum Yield, *J. Phys. Chem. C* **2010**, *114*, 18313.

[45] S. P. Schwaminger, D. Bauer, P. Fraga-Garcíagarcía, Gold-Iron Oxide Nanohybrids: Insights into Colloidal Stability and Surface-Enhanced Raman Detection, *Nanoscale*

Adv., **2021**, 3, 6438.
[46] J. Reguera, D. Jiménez de Aberasturi, N. Winckelmans, J. Langer, S. Bals, L. M. Liz-Marzán, Synthesis of Janus Plasmonic–Magnetic, Star–Sphere Nanoparticles, and Their Application in SERS Detection, *Faraday Discuss.* **2016**, *191*, 47.
[47] M. Talaikis, L. Mikoliunaite, A.-M. Gkouzi, V. Petrikaitė, E. Stankevičius, A. Drabavičius, A. Selskis, R. Juškėnas, G. Niaura, Multiwavelength SERS of Magneto-Plasmonic Nanoparticles Obtained by Combined Laser Ablation and Solvothermal Methods, *ACS Omega* **2023**, *8*, 49396.
[48] Y. Kang, X. Ye, J. Chen, L. Qi, R. E. Diaz, V. Doan-Nguyen, G. Xing, C. R. Kagan, J. Li, R. J. Gorte, E. A. Stach, C. B. Murray, Engineering Catalytic Contacts and Thermal Stability: Gold/Iron Oxide Binary Nanocrystal Superlattices for CO Oxidation, *J. Am. Chem. Soc.* **2013**, *135*, 1499.
[49] S. Tanaka, J. Lin, Y. V. Kaneti, S. Yusa, Y. Jikihara, T. Nakayama, M. B. Zakaria, A. A. Alshehri, J. You, M. S. A. Hossain, Y. Yamauchi, Gold Nanoparticles Supported on Mesoporous Iron Oxide for Enhanced CO Oxidation Reaction, *Nanoscale* **2018**, *10*, 4779.
[50] C.-T. Wang, S.-H. Ro, Surface Nature of Nanoparticle Gold/Iron Oxide Aerogel Catalysts, *J. Non. Cryst. Solids* **2006**, *352*, 35.
[51] T. Batsalova, A. Vasil'kov, D. Moten, A. Voronova, I. Teneva, A. Naumkin, B. Dzhambazov, Bimetallic Gold–Iron Oxide Nanoparticles as Carriers of Methotrexate: Perspective Tools for Biomedical Applications, *Appl. Sci.* **2023**, *13*, 12894.
[52] F. Pineider, C. De Julián Fernández, V. Videtta, E. Carlino, A. Al Hourani, F. Wilhelm, A. Rogalev, P. D. Cozzoli, P. Ghigna, C. Sangregorio, Spin-Polarization Transfer in Colloidal Magnetic-Plasmonic Au/Iron Oxide Hetero-Nanocrystals, *ACS Nano* **2013**, *7*, 857.
[53] A. Gabbani, E. Fantechi, G. Petrucci, G. Campo, C. de Julián Fernández, P. Ghigna, L. Sorace, V. Bonanni, M. Gurioli, C. Sangregorio, F. Pineider, Dielectric Effects in FeO x -Coated Au Nanoparticles Boost the Magnetoplasmonic Response: Implications for Active Plasmonic Devices, *ACS Appl. Nano Mater.* **2021**, *4*, 1057.
[54] A. L. Strickler, M. Escudero-Escribano, T. F. Jaramillo, Core–Shell Au@Metal-Oxide Nanoparticle Electrocatalysts for Enhanced Oxygen Evolution. *Nano Lett.* **2017**, *17*, 6040.
[55] S. Kang, A. Rahman, E. Boeding, P. J. Vikesland, Synthesis and SERS Application of Gold and Iron Oxide Functionalized Bacterial Cellulose Nanocrystals (Au@Fe 3 O 4 @BCNCs), *Analyst* **2020**, *145*, 4358.
[56] L. Mikoliunaite, E. Stankevičius, S. Adomavičiūtė-Grabusovė, V. Petrikaitė, R. Trusovas, M. Talaikis, M. Skapas, A. Zdaniauskienė, A. Selskis, V. Šablinskas, G. Niaura, Magneto-Plasmonic Nanoparticles Generated by Laser Ablation of Layered Fe/Au and Fe/Au/Fe Composite Films for SERS Application, *Coatings* **2023**, *13*, 1523.
[57] D. Shore, S. L. Pailloux, J. Zhang, T. Gage, D. J. Flannigan, M. Garwood, V. C. Pierre, B. J. H. Stadler, Electrodeposited Fe and Fe–Au Nanowires as MRI Contrast Agents, *Chem. Commun.* **2016**, *52*, 12634.
[58] S. Lu, X. Li, J. Zhang, C. Peng, M. Shen, X. Shi, Dendrimer-Stabilized Gold Nanoflowers Embedded with Ultrasmall Iron Oxide Nanoparticles for Multimode Imaging–Guided Combination Therapy of Tumors, *Adv. Sci.* **2018**, *5*.

[59] L. Bogani, L. Cavigli, C. de Julián Fernández, P. Mazzoldi, G. Mattei, M. Gurioli, M. Dressel, D. Gatteschi, Photocoercivity of Nano-Stabilized Au: Fe Superparamagnetic Nanoparticles, *Adv. Mater.* **2010**, *22*, 4054.

[60] C. de Julián Fernández, G. Mattei, E. Paz, R. L. Novak, L. Cavigli, L. Bogani, F. J. Palomares, P. Mazzoldi, A. Caneschi, C. Fernández, G. Mattei, E. Paz, R. L. Novak, L. Cavigli, L. Bogani, F. J. Palomares, P. Mazzoldi, A. Caneschi, Coupling between Magnetic and Optical Properties of Stable Au–Fe Solid Solution Nanoparticles, *Nanotechnology* **2010**, *21*, 165701.

[61] V. Amendola, S. Scaramuzza, L. Litti, M. Meneghetti, G. Zuccolotto, A. Rosato, E. Nicolato, P. Marzola, G. Fracasso, C. Anselmi, M. Pinto, M. Colombatti, Magneto-Plasmonic Au-Fe Alloy Nanoparticles Designed for Multimodal SERS-MRI-CT Imaging, *Small* **2014**, *10*, 2476.

[62] V. Torresan, D. Forrer, A. Guadagnini, D. Badocco, P. Pastore, M. Casarin, A. Selloni, D. Coral, M. Ceolin, M. B. Fernández van Raap, A. Busato, P. Marzola, A. E. Spinelli, V. Amendola, 4D Multimodal Nanomedicines Made of Nonequilibrium Au-Fe Alloy Nanoparticles, *ACS Nano* **2020**, *14*, 12840.

[63] F. Sousa, B. Sanavio, A. Saccani, Y. Tang, I. Zucca, T. M. Carney, A. Mastropietro, P. H. Jacob Silva, R. P. Carney, K. Schenk, A. O. Omrani, P. Huang, L. Yang, H. M. Rønnow, F. Stellacci, S. Krol, Superparamagnetic Nanoparticles as High Efficiency Magnetic Resonance Imaging T 2 Contrast Agent, *Bioconjug. Chem.* **2017**, *28*, 161.

[64] B. Sanavio, L. Librizzi, P. Pennacchio, G. V. Beznoussenko, F. Sousa, P. J. Silva, A. A. Mironov, C. Frassoni, F. Stellacci, M. de Curtis, S. Krol, Distribution of Superparamagnetic Au/Fe Nanoparticles in an Isolated Guinea Pig Brain with an Intact Blood Brain Barrier, *Nanoscale* **2018**, *10*, 22420.

[65] Y. Ruan, X. Wu, K. Li, J. Shen, J. Gong, K. Feng, S. Sun, X. Sun, FeAu Bimetallic Nanoparticle as Fe(0) Reservoir for Near Infrared Laser Enhanced Ferroptosis/Pyroptosis-Based Tumor Immunotherapy, *Adv. Funct. Mater.* **2024**, *34*, 2409335.

[66] Y. Li, M. Xu, U. Dhawan, W.-C. Liu, K.-T. Wu, X. Liu, C. Lin, G. Zhao, Y.-C. Wu, R.-J. Chung, Iron-Gold Alloy Nanoparticles Serve as a Cornerstone in Hyperthermia-Mediated Controlled Drug Release for Cancer Therapy, *Int. J. Nanomedicine* **2018**, *Volume 13*, 5499.

[67] U. Dhawan, C.-L. Tseng, P.-H. Wu, M.-Y. Liao, H.-Y. Wang, K. C.-W. Wu, R.-J. Chung, Theranostic Doxorubicin Encapsulated FeAu Alloy@metal-Organic Framework Nanostructures Enable Magnetic Hyperthermia and Medical Imaging in Oral Carcinoma. Nanomedicine Nanotechnology, *Nanomedicine Nanotechnology, Biol. Med.* **2023**, *48*, 102652.

[68] C.-K. Ting, U. Dhawan, C.-L. Tseng, C.-S. Alex Gong, W.-C. Liu, H.-D. Tsai, R.-J. Chung, Hyperthermia-Induced Controlled Local Anesthesia Administration Using Gelatin-Coated Iron–Gold Alloy Nanoparticles, *Pharmaceutics* **2020**, *12*, 1097.

[69] H.-H. Jeong, A. G. Mark, T.-C. Lee, M. Alarcón-Correa, S. Eslami, T. Qiu, J. G. Gibbs, P. Fischer, Active Nanorheology with Plasmonics, *Nano Lett.* **2016**, *16*, 4887.

[70] V. Amendola, S. Scaramuzza, S. Agnoli, S. Polizzi, M. Meneghetti, Strong Dependence of Surface Plasmon Resonance and Surface Enhanced Raman Scattering on the

Composition of Au-Fe Nanoalloys, *Nanoscale* **2014**, *6*, 1423.
[71] X. Liang, H. Wang, H. Wang, G. Pei, Colorimetric Detection of Bisphenol A Using Au–Fe Alloy Nanoparticle Aggregation, *Anal. Methods* **2015**, *7*, 3952.
[72] I. Vassalini, L. Borgese, M. Mariz, S. Polizzi, G. Aquilanti, P. Ghigna, A. Sartorel, V. Amendola, I. Alessandri,Enhanced Electrocatalytic Oxygen Evolution in Au–Fe Nanoalloys, *Angew. Chemie Int. Ed.* **2017**, *56*, 6589.
[73] X. Li, Y. Luo, S. Wu, H. Lian, X. Deng, The Exceptional Performance of the Plasmonic Au-Fe/TiO2 Nanocatalysts Achieved by O2 Plasma Activation, *Catal. Today* **2023**, *418*, 114106.
[74] G. M. Spataro, J. Yang, V. Coviello, S. Agnoli, V. Amendola, Surface Gold Atoms Determine Peroxidase Mimic Activity in Gold Alloy Nanoparticles, *ChemPhysChem* **2024**, *25*, e202400486.
[75] A. Tymoczko, M. Kamp, C. Rehbock, L. Kienle, E. Cattaruzza, S. Barcikowski, V. Amendola, One-Step Synthesis of Fe-Au Core-Shell Magnetic-Plasmonic Nanoparticles Driven by Interface Energy Minimization, *Nanoscale Horizons* **2019**, *4*, 1326.
[76] E. Tiryaki, T. Zorlu, R. A. Alvarez-Puebla, Magnetic–Plasmonic Nanocomposites as Versatile Substrates for Surface–Enhanced Raman Scattering (SERS) Spectroscopy, *Chem. – A Eur. J.* **2024**, *30*.
[77] K. Sun, T. Cheng, L. Wu, Y. Hu, J. Zhou, A. Maclennan, Z. Jiang, Y. Gao, W. A. Goddard, Z. Wang, Ultrahigh Mass Activity for Carbon Dioxide Reduction Enabled by Gold–Iron Core–Shell Nanoparticles, *J. Am. Chem. Soc.* **2017**, *139*, 15608.
[78] P. Wagener, J. Jakobi, C. Rehbock, V. S. K. Chakravadhanula, C. Thede, U. Wiedwald, M. Bartsch, L. Kienle, S. Barcikowski, Solvent-Surface Interactions Control the Phase Structure in Laser-Generated Iron-Gold Core-Shell Nanoparticles, *Sci. Rep.* **2016**, *6*, 23352.
[79] A. Tymoczko, M. Kamp, O. Prymak, C. Rehbock, J. Jakobi, U. Schürmann, L. Kienle, S. Barcikowski, How the Crystal Structure and Phase Segregation of Au–Fe Alloy Nanoparticles Are Ruled by the Molar Fraction and Size, *Nanoscale* **2018**, *10*, 16434.
[80] V. Coviello, D. Badocco, P. Pastore, M. Fracchia, P. Ghigna, A. Martucci, D. Forrer, V. Amendola, Accurate Prediction of the Optical Properties of Nanoalloys with Both Plasmonic and Magnetic Elements, *Nat. Commun.* **2024**, *15*, 834.
[81] M. Kamp, A. Tymoczko, R. Popescu, U. Schürmann, R. Nadarajah, B. Gökce, C. Rehbock, D. Gerthsen, S. Barcikowski, L. Kienle, . Composition and Structure of Magnetic High-Temperature-Phase, Stable Fe-Au Core-Shell Nanoparticles with Zero-Valent Bcc Fe Core, *Nanoscale Adv.* **2020**, *2*, 3912.
[82] S. Scaramuzza, S. Agnoli, V. Amendola, Metastable Alloy Nanoparticles, Metal-Oxide Nanocrescents and Nanoshells Generated by Laser Ablation in Liquid Solution: Influence of the Chemical Environment on Structure and Composition, *Phys. Chem. Chem. Phys.* **2015**, *17*, 28076.
[83] A. Tymoczko, M. Kamp, C. Rehbock, L. Kienle, E. Cattaruzza, S. Barcikowski, V. Amendola, One-Step Synthesis of Fe–Au Core–Shell Magnetic-Plasmonic Nanoparticles Driven by Interface Energy Minimization, *Nanoscale Horiz.* **2019**, *4*, 1326.
[84] G. Yang, D. V Mamonova, A. A. Vasileva, Y. V Petrov, A. V Koroleva, D. V Danilov, I. E. Kolesnikov, G. I. Bikbaeva, J. Bachmann, A. A. Manshina, Single Step Laser-

Induced Deposition of Plasmonic Au, Ag, Pt Mono-, Bi- and Tri-Metallic Nanoparticles, *Nanomater. 2022, Vol. 12, Page 146* **2021**, *12*, 146.

[85] H. Fuse, N. Koshizaki, Y. Ishikawa, Z. Swiatkowska-Warkocka, H. Fuse, N. Koshizaki, Y. Ishikawa, Z. Swiatkowska-Warkocka, Determining the Composite Structure of Au-Fe-Based Submicrometre Spherical Particles Fabricated by Pulsed-Laser Melting in Liquid, *Nanomaterials* **2019**, *9*, 198.

[86] J. Zhang, M. Post, T. Veres, Z. J. Jakubek, J. Guan, D. Wang, F. Normandin, Y. Deslandes, B. Simard, Laser-Assisted Synthesis of Superparamagnetic Fe@Au Core−Shell Nanoparticles, *J. Phys. Chem. B* **2006**, *110*, 7122.

[87] C. Srivastava, S. Chithra, K. D. Malviya, S. K. Sinha, K. Chattopadhyay, Size Dependent Microstructure for Ag–Ni Nanoparticles, *Acta Mater.* **2011**, *59*, 6501.

[88] W. T. Osowiecki, X. Ye, P. Satish, K. C. Bustillo, E. L. Clark, A. P. Alivisatos, Tailoring Morphology of Cu–Ag Nanocrescents and Core–Shell Nanocrystals Guided by a Thermodynamic Model, *J. Am. Chem. Soc.* **2018**, *140*, 8569.

[89] J. Johny, M. Kamp, O. Prymak, A. Tymoczko, U. Wiedwald, C. Rehbock, U. Schürmann, R. Popescu, D. Gerthsen, L. Kienle, S. Shaji, S. Barcikowski, Formation of Co-Au Core-Shell Nanoparticles with Thin Gold Shells and Soft Magnetic ϵ-Cobalt Cores Ruled by Thermodynamics and Kinetics, *J. Phys. Chem. C* **2021**, *125*, 9534.

[90] I. Barin, O. Knacke, O. Kubaschewski, *Thermochemical properties of inorganic substances*, Springer Berlin Heidelberg, Berlin, Heidelberg **1977**.

[91] H. Okamoto, T. B. Massalski, L. J. Swartzendruber, P. A. Beck, The Au−Fe (Gold-Iron) System, *Bull. Alloy Phase Diagrams* **1984**, *5*, 592.
